# Focal Transplantation of Human iPSC-Derived Glial-Rich Neural Progenitors Improves Lifespan of ALS Mice

**DOI:** 10.1016/j.stemcr.2014.05.017

**Published:** 2014-06-26

**Authors:** Takayuki Kondo, Misato Funayama, Kayoko Tsukita, Akitsu Hotta, Akimasa Yasuda, Satoshi Nori, Shinjiro Kaneko, Masaya Nakamura, Ryosuke Takahashi, Hideyuki Okano, Shinya Yamanaka, Haruhisa Inoue

**Affiliations:** 1 Center for iPS Cell Research and Application (CiRA), Kyoto University, Kyoto 606-8507, Japan; 2 Department of Neurology, Graduate School of Medicine, Kyoto University, Kyoto 606-8507, Japan; 3 CREST, JST, Saitama 332-0012, Japan; 4 PRESTO, JST, Saitama 332-0012, Japan; 5 iCeMS, Kyoto University, Kyoto 606-8507, Japan; 6 Department of Orthopedic Surgery, School of Medicine, Keio University, Tokyo 160-8582, Japan; 7 Department of Orthopaedic Surgery, National Hospital Organization, Murayama Medical Center, Tokyo 208-0011, Japan; 8 Department of Physiology, School of Medicine, Keio University, Tokyo 160-8582, Japan; 9 Gladstone Institute of Cardiovascular Disease, San Francisco, CA 94158, USA

## Abstract

Transplantation of glial-rich neural progenitors has been demonstrated to attenuate motor neuron degeneration and disease progression in rodent models of mutant superoxide dismutase 1 (SOD1)-mediated amyotrophic lateral sclerosis (ALS). However, translation of these results into a clinical setting requires a renewable human cell source. Here, we derived glial-rich neural progenitors from human iPSCs and transplanted them into the lumbar spinal cord of ALS mouse models. The transplanted cells differentiated into astrocytes, and the treated mouse group showed prolonged lifespan. Our data suggest a potential therapeutic mechanism via activation of AKT signal. The results demonstrated the efficacy of cell therapy for ALS by the use of human iPSCs as cell source.

## Highlights

• Transplantation of human iPSC-derived cells to spinal cord of ALS model mice
• Transplanted glial-rich NPCs attenuated non-cell autonomous neurodegeneration
• Feasibility study for ALS transplantation is presented

## Graphical Abstract

**Figure:**
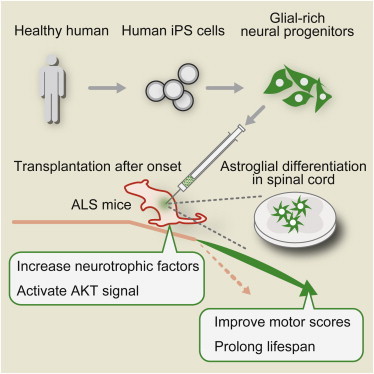

## Teaser

Transplantation research as ALS therapy is an important issue. In this study, Inoue and colleagues transplanted glial-rich neural progenitors derived from human iPSCs into the lumbar spinal cord of ALS mice. The transplanted cells differentiated into astrocytes, and the treated mouse group showed prolonged lifespan via increased neurotrophic factors and activation of AKT signal. Human iPSCs could be a promising resource for ALS transplantation therapy.

## Introduction

Amyotrophic lateral sclerosis (ALS) is a disorder of motor neurons (MNs) that is characterized by their relatively rapid degeneration, resulting in progressive muscle weakness and respiratory failure (Bruijn et al., 2004). Approximately 90%–95% of ALS cases are sporadic in nature, with 20% of the remaining familial cases linked to various point mutations in the Cu/Zn superoxide dismutase 1 (SOD1) gene. Transgenic mice and rats carrying ALS-associated mutant human SOD1 genes (mSOD1) recapitulate many features of the human disease (Gurney et al., 1994).

Despite the relative selectivity of MN loss in ALS, studies in mSOD1 rodent and tissue culture models show nonneuronal (glial) cell involvement in the disease process (Boillée et al., 2006; Yamanaka et al., 2008). Astrocytes in particular are hypothesized to play a role in both mSOD1 and sporadic forms of ALS (Haidet-Phillips et al., 2011; Howland et al., 2002; Papadeas et al., 2011). Regardless of whether astrocyte dysfunction is a cause of the disease or a consequence of neuronal death, altered astrocyte physiology results in further susceptibility to MN loss (Boillée et al., 2006). Targeted enrichment of normal astrocytes in mSOD1 rat spinal cord via intraspinal transplantation of rodent glial-restricted progenitors promoted focal MN protection, delayed decline in respiratory function, and extended disease progression (Xu et al., 2011).

Various kinds of cells have been investigated for transplantation studies (Corti et al., 2004; Garbuzova-Davis et al., 2008; Iwanami et al., 2005; Piccini et al., 1999). Neuronal cells are probably the most relevant cell type for ALS treatment, but such cells suffer from a limited supply, ethical issues, and/or invasive harvest from human donors. On the other hand, human induced pluripotent stem cells (hiPSCs) can be obtained from a donor less invasively and can be expanded indefinitely in vitro. In this context, here we established a differentiation protocol of glial-rich neural progenitors (GRNPs) from hiPSCs and investigated the potential of hiPSC-derived glial-rich neural progenitors (hiPSC-GRNPs) as a cell source for intraspinal transplantation therapy of ALS.

## Results

### Cell Resource Establishment for Transplantation

**Figure 1:**
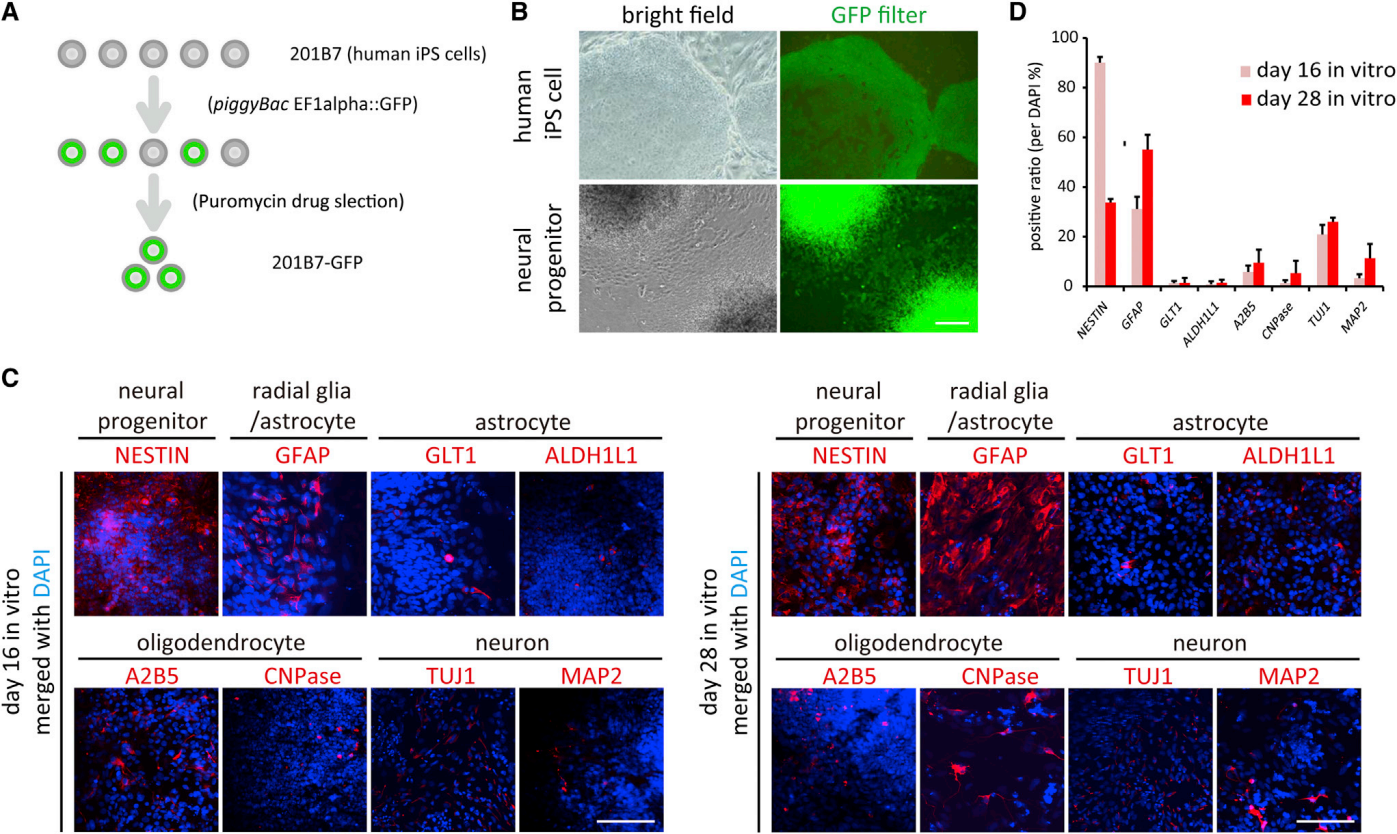
Human iPSCs Were Labeled with GFP, and They Differentiated into Neural Precursors (A) hiPSCs were labeled with GFP by a *piggyBac* vector. (B) GFP-labeled hiPSCs retained GFP signals after neural induction. (C) hiPSC-derived neural precursors exhibited immunoreactivities for NESTIN (neural precursor marker), GFAP (astroglial or radial-glial marker), GLT1/ALDH1 (functional/mature astrocyte marker), A2B5/CNPasae (oligodendrocyte lineage marker), and TUJ1/MAP2 (neural lineage marker). (D) Quantification of hiPSC differentiation in (C). Data represent mean ± SD (n = 3 experiments). Scale bars, 200 μm.

As a cell resource, we selected human iPSC line “201B7 clone,” which had been previously evaluated as possessing low tumorigenicity after transplantation therapy (Kobayashi et al., 2012; Nori et al., 2011) To distinguish the transplanted cells from host cells, we introduced a *piggyBac* vector, which stably expresses GFP gene under the control of the ubiquitous EF1α promoter, into hiPSCs and observed continuous GFP fluorescence even after neural-lineage differentiation (Figures 1A and 1B). We differentiated GFP-labeled hiPSCs into neural stem cells by the serum-free floating culture of embryoid bodies-like aggregates method with SMAD-pathway inhibition (Kondo et al., 2013). Neural stem cells were efficiently differentiated into hiPSC-GRNPs by stimulation of the LIF/BMP signaling. This protocol provided highly enriched neural precursors, 68.4% ± 7.2% positive for NESTIN and 54.9% ± 6.1% positive for GFAP (Figures 1C and 1D). At day 16 in vitro, most of the differentiated grafts were positive for NESTIN or GFAP. At this very early stage, GFAP^+^ cells include either radial glia, a subtype of developmental neural progenitors with a neuron-like spine, or immature astrocytes (Liour and Yu, 2003). At day 28 in vitro, NESTIN^+^ neural progenitors differentiated into TUJ1^+^ neurons, A2B5^+^ oligoprogenitors, and GFAP^+^ astrocytes. The differentiation method used in the present work could augment the GFAP^+^ glial population and attenuate TUJ1 neural differentiation, as compared with our previous method (Kondo et al., 2013). However, GFAP^+^ astrocytes were not positive for GLT1 or ALDH1L1, which were thought to be functionally mature astrocytes before transplantation.

### hiPSC-GRNPs Transplantation Improved Motor Function and Survival in ALS Model Mice

**Figure 2:**
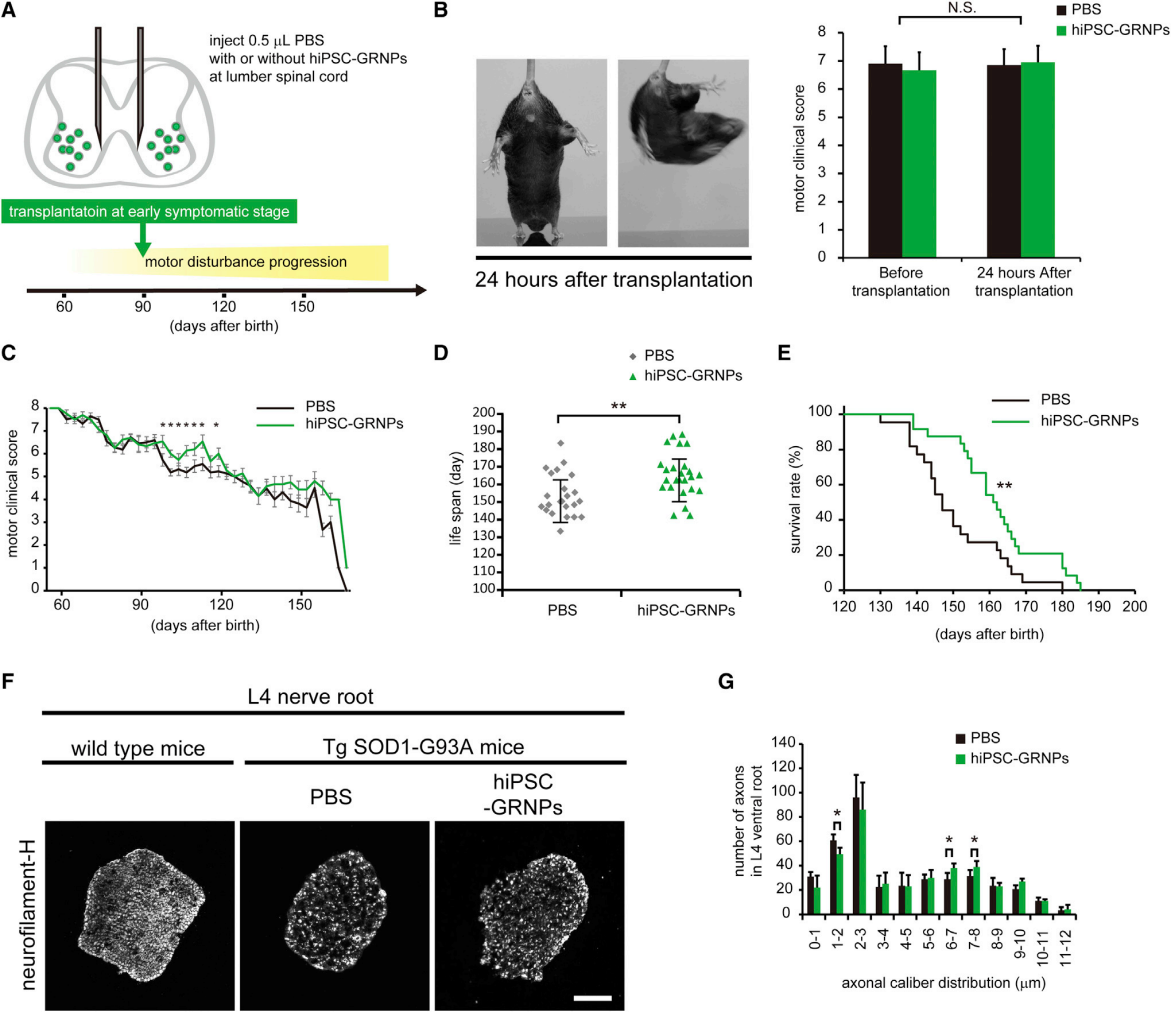
hiPSC-GRNPs Transplantation Improved Motor Score and Survival in ALS Model Mice (A) Transplantation schedule and schema of spinal cord injection site. Transplantation was performed after disease onset. (B) Mice presented with no side effects after transplantation and made vigorous twisting movements with hind-limb extension, as shown by representative photos at tail suspension. Both groups showed no change in motor clinical score at 24 hr after surgical insult. (C) Clinical motor scoring change by sequential evaluation showed significant difference from 100 to 120 days after birth (^∗^p < 0.05). Data represent mean ± SEM (n = 21 mice per group). (D) Lifespan was prolonged in the hiPSC-GRNPs transplantation group (162.2 ± 12.8 days) compared to the control group (150.4 ± 12.1 days) (^∗∗^p < 0.01). Data represent mean ± SD (n = 21 mice per group). (E) Survival (Kaplan-Meier plot) analysis shows a significant difference between the PBS injection group survival (black line) and the hiPSC-GRNPs transplantation group survival (green line) throughout the course of the study (n = 21 mice per group, p = 0.00691 stratified log-rank test), suggesting that the hiPSC-GRNPs transplantation group had better survival (^∗∗^p < 0.01). (F) The number of axons in L4 ventral nerve root was counted to estimate surviving motor neurons at the middle stage of disease progression. At 120 days after birth, transverse sections of ventral nerve roots were stained with an anti-neurofilament-H antibody. Compared to littermates without transgene, the number of axons in Tg SOD1-G93A mice was decreased. Each axon caliber was measured and classified according to size. (G) Cumulative axon caliber distribution at L4 ventral root at 120 days after birth of both groups. Two-way ANOVA with repeated-measures was used to study the effect of transplantation (transplanted and nontransplanted mice) on axonal caliber distribution. Pairwise comparisons were made using Bonferroni adjustment (^∗^p < 0.05). The number of 1–2 μm caliber axons was significantly decreased and the number of 6–8 μm caliber axons was significantly increased in the hiPSC-GRNPs transplantation group. Data represent mean ± SD (n = 3 mice per group). Scale bars, 100 μm. hiPSCs, human induced pluripotent stem cells; hiPSC-GRNPs, hiPSC-derived glial-rich neural progenitors. See also Figure S1 and Table S1.

All animal experiments were approved by the CiRA Animal Experiment Committee (nos. 24 and 27). We transplanted 40,000 hiPSC-derived GRNPs each into bilateral lumbar spinal cords of transgenic SOD1-G93A mice. Transplantation operations were performed after onset of ALS phenotype, at 90 days of age to mimic the clinical situation (Figure 2A). Littermates of transplanted mice received only a vehicle (PBS) injection and were used as control group. We designed the study so that siblings were distributed equally in the control (n = 24, male:female = 17:7) and transplanted (n = 24, male:female = 17:7) groups. By using a 35 gauge needle and a relatively small injection volume, we could avoid motor disturbance at 24 hr after the surgical insult (Figure 2B), evaluated by clinical grading system (Table S1 available online).

At 10–40 days after the procedure, we observed improvement in clinical motor score in the hiPSC-GRNPs transplantation group (Figure 2C). Surviving lifespan was extended by 7.8% in the hiPSC-GRNPs transplantation group (n = 21, male:female = 14:7, 162.2 ± 12.8 days) compared to the control group (n = 21, male:female = 14:7, 150.4 ± 12.1 days) (Figures 2D and 2E). When the effect of transplantation was evaluated separately in male and female mice, a greater survival improvement was noted in males than in females (Figure S1). Survival lifespan was significantly expanded only in males (145.3 ± 9.8 days for control, 158.5 ± 11.2 days for the hiPSC-GRNPs transplantation group, extended by 9.1%), not in females (160.5 ± 9.5 days for control, 169.7 ± 12.6 days for the hiPSC-GRNPs transplantation group, extended by 5.7%). To evaluate motor neuron degeneration at the symptomatic phase, three male mice from each group were sacrificed at 120 days of age. The number of 6–8 μm large-caliber fibers was increased in the hiPSC-GRNPs transplantation group (Figures 2F and 2G). We could not detect GFP signals, derived from transplanted hiPSC-GRNPs, in nerve root slices.

### Transplanted hiPSC-GRNPs Differentiated into Astrocytes in Spinal Cord of ALS Model Mice without Tumorigenic Formation

**Figure 3:**
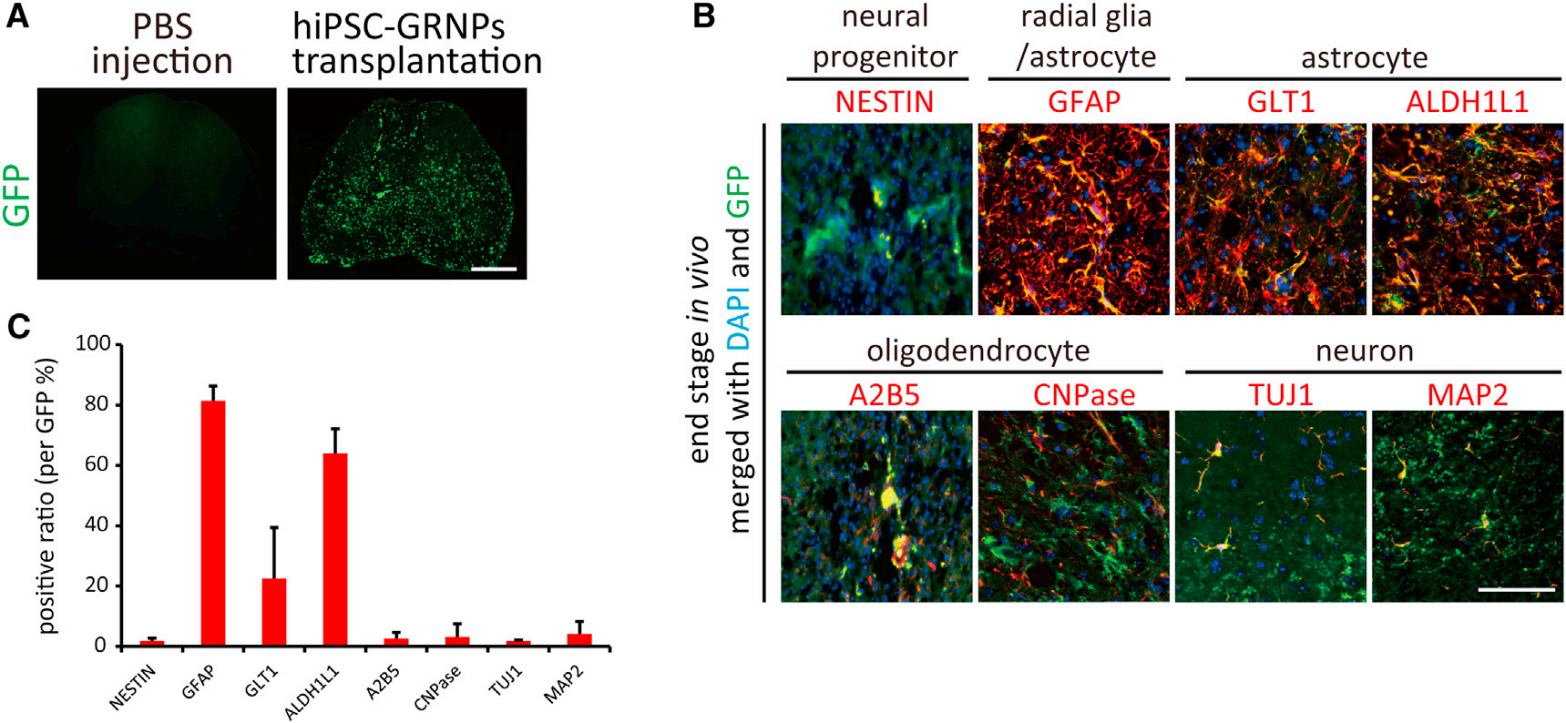
Transplanted Cells Differentiated Mainly into Astrocytes (A) Transplanted GFP-labeled grafts (green) survived in the spinal cord. Scale bars, 500 μm. (B) Most of the GFP-labeled grafts in ventral horn parenchyma differentiated into mature astrocytes, which were positive for astrocyte markers (red), including GFAP, ALDH1L1, and GLT1, in vivo. A relatively small population of grafts also differentiated into CNPase^+^ oligodendrocytes or MAP2^+^ neurons. A limited number of grafts remained as NESTIN^+^ neural progenitors. Scale bar, 50 μm. (C) Quantification of positive ratio of cell type markers in (B). A small number of GFP-labeled grafts were also positive for A2B5, an oligoprogenitor marker, MAP2, a neuronal marker, or Nestin, a neural precursor marker, in vivo. The total number of GFP-positive cells, counted to calculate the positive ratio, was 113.3 ± 14.7 for NESTIN, 75.9 ± 11.8 for GFAP, 68.7 ± 5.5 for GLT1, 76.9 ± 9.4 for ALDH1L1, 67.3 ± 13.0 for A2B5, 69.3 ± 19.2 for CNPase, 70 ± 13.6 for TUJ1, and 66.7 ± 20.2 for MAP2, respectively. Data represent mean ± SD (n = 3 mice per group). See also Figure S2.

We continued to evaluate clinical motor function (Table S1) and defined clinical grade 0 as end stage. At the end stage of disease progression, around 140–170 days after birth, the animals were sacrificed and histological analysis was performed to investigate the state of the engraftment. Transplanted hiPSC-GRNP-derived cells, which are positive for GFP signals, continued to survive in the lumbar spinal cord of ALS model mice (Figure 3A). Engraftment could be observed at least 5 mm away from the injection site. We assessed some cell subtype markers by immunostaining, including GFAP/GLT1/ALDH1L1 for astrocytes (Figure 3B), A2B5 for oligodendroglial progenitors, and NESTIN for neural progenitors (Figure 3C). Around 60%–80% of the cells at the grafts were double-positive for GFP fluorescence and GFAP marker, suggesting that transplanted hiPSC-GRNPs had differentiated into astrocytes (Figure 3C). Although we hardly observed the immunoreactivity of functional/mature astrocyte markers including GLT1 and ALDH1L1 in vitro before transplantation, we did observe it after transplantation. The rate of neurons or oligodendrocytes was low (Figure 3C). Only a small population of grafts retained positive staining for NESTIN, that is, remaining in the neural progenitor stage of differentiation (Figure 3C).

It is important to note that, during our observation period (up to 3 months posttransplantation), the injection sites showed no signs of tumor formation. Gross pathological examinations of other organs outside the CNS did not reveal any heterotopic engraftment.

### Transplanted hiPSC-GRNPs Upregulated Neurotrophic Factors and Activated Cell Survival Signal

**Figure 4:**
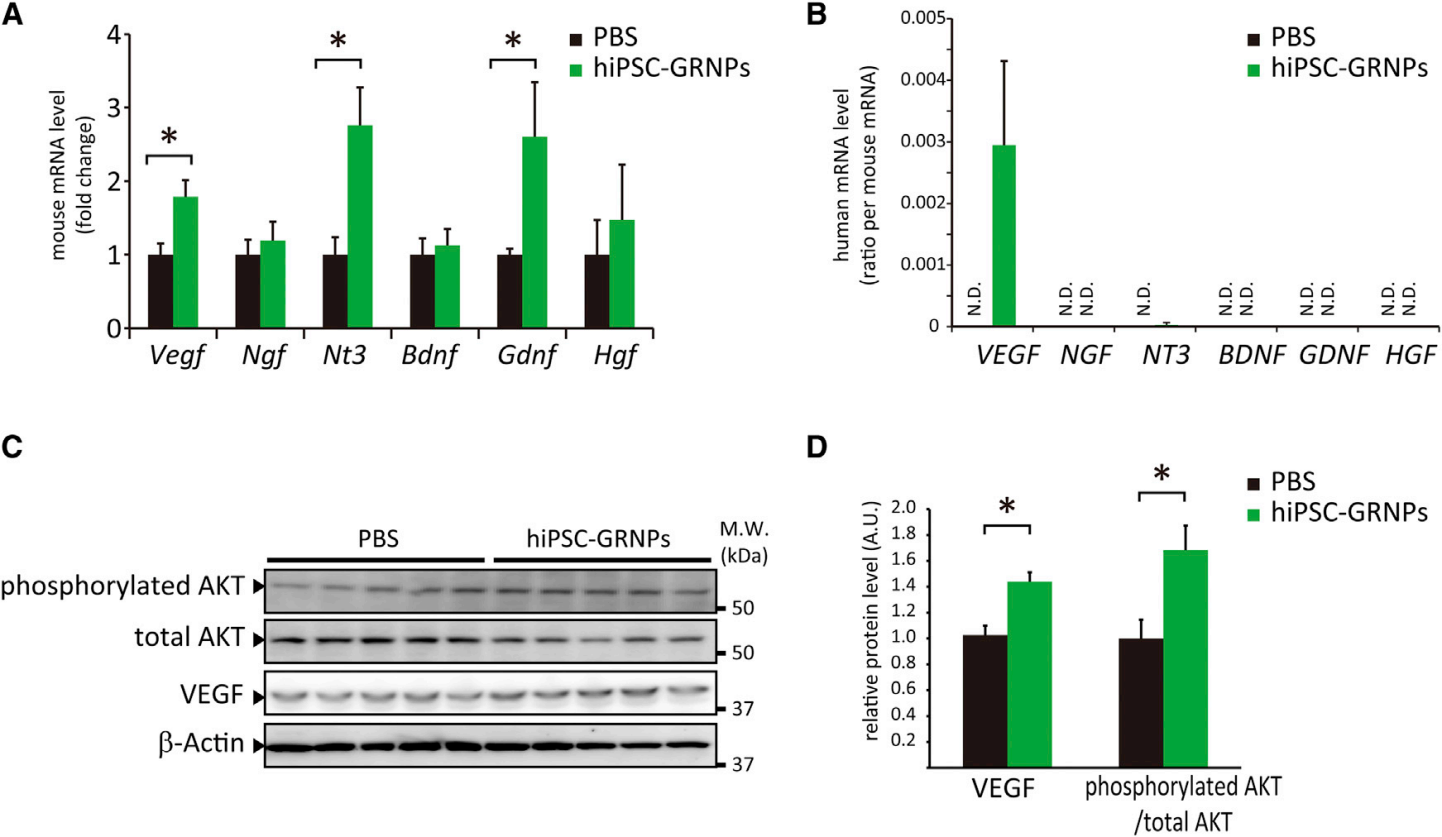
hiPSC-GRNPs Transplantation Increased Neurotrophic Factors and Activated AKT Signal (A) Gene expressions of neurotrophic factors, including *Vegf*, *Ngf*, *Nt3*, *Bdnf*, *Gdnf*, and *Hgf* were quantitatively analyzed using mouse-specific primers. The levels of *Vegf*, *Nt3*, and *Gdnf* were significantly increased in the hiPSC-GRNPs transplantation group (^∗^p < 0.05). Data represent mean ± SD (n = 3 mice per group). (B) Gene expressions of neurotrophic factors, including *VEGF*, *NGF*, *NT3*, *BDNF*, *GDNF*, and *HGF* were quantitatively analyzed using human-specific primers and the human-origin/mouse-origin ratio was calculated. Data represent mean ± SD (n = 3 mice per group). (C) Western blot analysis of phosphorylated AKT, total AKT level, and VEGF in lumbar spinal cord in the PBS injection group and hiPSC-GRNPs transplantation group. (D) Densitometric analysis of (C). Measured values of proteins were normalized by that of β-actin. The levels of VEGF and AKT phosphorylation were significantly increased in the hiPSC-GRNPs transplantation group (^∗^p < 0.05). Data represent mean ± SD (n = 5 mice per group). N.D., not detectable. See also Figure S3 and Table S2.

We investigated the expression level of neurotrophic factors in lumbar spinal cord. We designed mouse- or human-specific primers to evaluate host- or graft-derived mRNA separately (Table S2). Quantitative RT-PCR revealed that upregulated expressions were observed in mouse (host)-originated *Vegf*, *Nt3*, and *Gdnf*, but not in *Ngf*, *Bdnf*, or *Hgf* (Figure 4A). However, human (graft)-originated expression, equal to hiPSC-GRNPs origin, was observed only in *VEGF* (Figure 4B). Western blot analysis demonstrated a significant increase in VEGF level in the hiPSC-GRNPs transplantation group (Figures 4C and 4D). Furthermore, hiPSC-GRNPs transplantation increased phosphorylated AKT and activated AKT signaling, which is downstream from the VEGF signal and is important for cell survival in ALS (Lunn et al., 2009) (Figures 4C and 4D).

## Discussion

Here, we describe that transplantation of human iPSC-derived GRNPs produced astrocytes in vivo and prolonged the survival period of mSOD1 mice. We used hiPSC-GRNPs for testing the efficacy in mSOD1 mice, because replacement therapy using astrocytes from rodent glial-restricted progenitors in the cervical spinal cord of ALS rodent models is already well established (Xu et al., 2011).

We showed that glial cells represent a potential target of ALS therapy. However, we observed transient improvement of lower limb function, as shown in Figure 2C, and a similar previous study failed to show improvement in the rescue of clinical manifestations and neuronal survival by transplantation of human-derived glial-restricted progenitor cells from 17- to 24-week fetus into SOD1 transgenic mouse spinal cord despite the survival and proliferation of exogenous astrocytes (Lepore et al., 2011). Although the transient improvement in our study might have stemmed from neuroprotective effects of the transplanted cells only in the lumbar region, with a possible broader effect of neurotrophic factors on other regions or behavioral alteration for food intake, as previously discussed (Table S1), we comprehensively compared the two studies as well as others regarding lumbar transplantation in terms of a number of aspects (Table S1), speculating that there were differences in graft type, transplantation condition, and/or transplantation timing. Regarding the timing of transplantation, the survival improvement in our study might have resulted in attenuation of the glial contribution to the disease pathogenesis at an early symptomatic stage (Boillée et al., 2006; Yamanaka et al., 2008). Regarding the cell injection site, instead of the cervical cord, we injected the cells into the lumbar spinal cords of ALS model mice, which resulted in improved clinical scores of lower limbs. These data supported the possibility of targeting not only the cervical cord but also the lumbar spinal cord in ALS clinical trials, depending on the symptoms to be treated. Following previous transplantation research (Table S1), we selected PBS, which is a vehicle solution for grafts, as control agent of transplantation. Dead cells or fibroblasts can be appropriate control agents but may also possibly secrete various factors. Furthermore, previously an extensive study showed that, as a control agent, there was no significant difference among vehicle solution, dead cells, and fibroblasts (Lepore et al., 2008).

iPSCs were previously reported to induce T cell-dependent immune response by direct transplantation of undifferentiated cells into syngeneic mice. However, a more detailed investigation proved that autologous transplantation of terminally differentiated cells derived from iPSCs or embryonic stem cells elicits only negligible immunogenicity (Araki et al., 2013; Okano et al., 2013). In our study, we did not observe excess inflammatory responses around transplanted cells under treatment of low-dose immunosuppression, suggesting that even if transplanted cells were not autologous, we could control the immune responses of the recipients by immunosuppressant treatment.

The increase in the levels of neurotrophic factors had been commonly observed in transplantation therapy of ALS models (Nizzardo et al., 2014; Teng et al., 2012). We observed that the transplanted hiPSC-GRNPs produced VEGF, and expressions of endogenous VEGF and other neurotrophic factors in the host mice were upregulated. A previous study showed that VEGF retrograde delivery with lentiviral vector could prolong the survival of ALS model mice by 30% (Azzouz et al., 2004) and that activated AKT signaling, which is downstream of VEGF, is important for cell survival in ALS (Lunn et al., 2009). Similarly in this study, transplanted hiPSC-GRNPs increased the VEGF level and prolonged the survival of mSOD1 mice. We could observe positive immunostaining for VEGF and phosphorylated AKT in both remaining motor neurons and astrocytes. However, we could not observe any morphological difference in motor neurons between control and transplanted groups at end-stage. We also speculated that, as shown in previous studies (Howland et al., 2002), transplanted hiPSC-GRNPs differentiated into astrocytes expressing glutamate transporter 1 (GLT1) might restore glutamate homeostasis in our study.

In our study, the males in both the control and hiPSC-GRNPs transplantation groups died long before the females, and this result is consistent with the previous reports of ALS model mice (Cervetto et al., 2013; Choi et al., 2008). However, improvement in male mice was greater than in females (Figure S1) Interestingly, a similar gender-dependent difference of therapeutic efficacy was reported in ALS model mice (Cervetto et al., 2013; Li et al., 2012). The epidemiological studies of sporadic ALS have shown that both incidence and prevalence of ALS are greater in men than in women and onset of the disease is also earlier for men than it is for women (McCombe and Henderson, 2010). Sex steroids are suggested to be involved in the gender difference in ALS, but the direct importance of estrogen is still controversial (Choi et al., 2008; Li et al., 2012). Moreover, male neural cells are reported to be more vulnerable to oxidative stress, induced by mutant SOD1 overexpression, than female neural cells (Li et al., 2012). In our study, transplanted cells were mainly differentiated into GFAP-positive astrocytes and upregulated VEGF. Furthermore, astrocytes can play neuroprotective roles from oxidative stress via VEGF (Chu et al., 2010). These findings suggest that hiPSC-GRNPs transplantation may ameliorate male-specific vulnerability to oxidative stress and improve the survival lifespan of male mice. Further analysis would be necessary to elucidate VEGF-associated mechanisms in transplantation therapy.

In regard to safety, the potential tumorigenicity of grafts is a predominant concern. We used the human iPSC line “201B7,” which was previously reported to be safe from the viewpoint of tumorigenesis (Kobayashi et al., 2012). Furthermore, we found no signs of tumor formation or Ki67-positive grafts (Figure S2). However, a very small proportion of grafts remained positive for the neural progenitor marker NESTIN at 3 months posttransplantation. We cannot exclude the risk of tumor formation from the remaining NESTIN-positive NPCs. It is important to evaluate tumorigenicity by longer-term observations for future clinical trials.

We tested the potential of cell therapy after onset of the disease in ALS model mice, because most human cases of ALS are sporadic and any treatment would be initiated after onset. Our study showed a modest lifespan prolongation compared to previous studies testing cell therapy before disease onset in ALS model mice. Future studies of transplantations, such as combinations with MN engraftment, will be required to accelerate ALS treatment toward restoration of MN function and ultimately the complete cure of ALS.

## Experimental Procedures

### Preparation of hiPSC-GRNPs for Transplantation

We differentiated hiPSCs into neural lineage cells using a previously described differentiation protocol (Kondo et al., 2013), under the condition of additional 10 ng/ml human BMP4 (R&D Systems) and 10 ng/ml human LIF (R&D) during the patterning stage (days 8–28).

### Transplantation

We transplanted 40,000 hiPSC-GRNPs into bilateral lumbar spinal cords of 90-day-old Tg SOD1-G93A mice. Each mouse received two grafts (bilaterally at L3-L4) of 4 × 10^4^ cells (in 0.5 μl PBS) into the ventral horn.

### Statistical Analysis

The Mann-Whitney test was used for the analysis of two populations of means, and p values <0.05 were considered significant. Repeated-measures two-way ANOVA, followed by the Tukey-Kramer test, was used for clinical motor scoring analysis. The Kaplan-Meier plot was used to evaluate survival time, and the log-rank test was applied to compare cumulative curves.

## Author Contributions

H.I. conceived the project; T.K. and H.I. designed the experiments; T.K. M.F., K.T., A.Y., and S.N. performed the experiments; T.K., M.N., H.O., and H.I. analyzed the data; A.H. and S.Y. contributed reagents, materials, and analysis tools; A.Y., S.N., S.K., M.N., R.T., and H.O. provided critical reading and scientific discussions; T.K., A.H., H.O., and H.I. wrote the paper.

## Supplemental Information

Document S1. Supplemental Experimental Procedures, Figures S1–S3, and Tables S1 and S2

Document S2. Article plus Supplemental Information
