# Supplementary material for: Focal Transplantation of Human iPSC-Derived Glial-Rich Neural Progenitors Improves Lifespan of ALS Mice
Source: Stem Cell Reports. 2014 Jun 26;3(2):242–9. doi: 10.1016/j.stemcr.2014.05.017 (PMC4175543; doi:10.1016/j.stemcr.2014.05.017)
Supplement: Document S1. Supplemental Experimental Procedures, Figures S1–S3, and Tables S1 and S2 [file mmc1.pdf]

Stem Cell Reports, Volume *3*

Supplemental Information

Focal Transplantation of Human iPSC-Derived Glial-Rich Neural Progenitors Improves Lifespan of ALS Mice

Takayuki Kondo, Misato Funayama, Kayoko Tsukita, Akitsu Hotta, Akimasa Yasuda, Satoshi Nori, Shinjiro Kaneko, Masaya Nakamura, Ryosuke Takahashi, Hideyuki Okano, Shinya Yamanaka, and Haruhisa Inoue

Figure S1

A

Grade	Clinical manifestation
8	Normal functions, no sign of disease onset
7	Hind limb tremors when suspended by the tail
6	Weakness of one hindlimb extension when suspended by the tail
5	Weakness of two hindlimbs extension when suspended by the tail
4	One proximal hindlimb paralysed and walking on tiptoes
3	Two proximal hindlimbs paralysed and walking on tiptoes
2	Dragging one rigid hind limb
1	Dragging two rigid hind limbs
0	Unable to right itself within 30 s

B

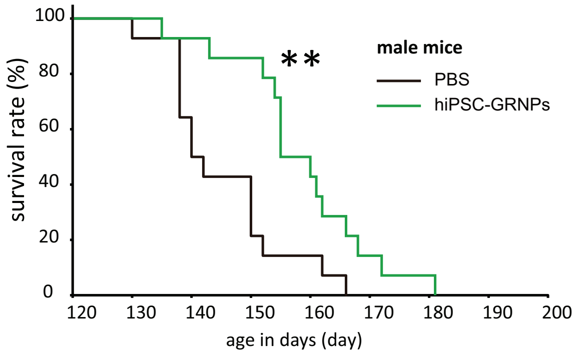

C

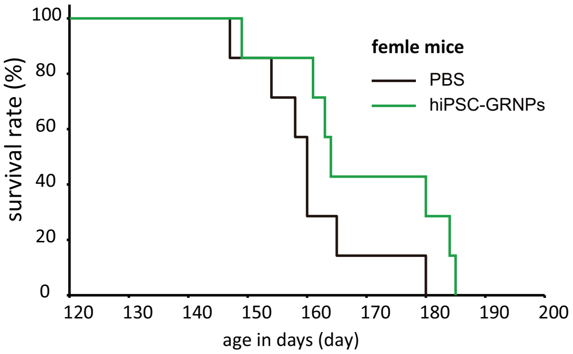

Figure S1, related to Figure 2. Separate evaluations of survival of male and female mice

(A) Motor function scoring system. (B) Kaplan-Meier plot of male ALS mice shows a significant difference between the PBS injection group survival (black line) and the hiPSC-GRNPs transplantation group survival (green line) throughout the course of the study (n = 14 mice per group, P = 0.00479 stratified log-rank test), suggesting that the hiPSC-GRNPs transplantation group had better survival (**p < 0.01). (C) Kaplan-Meier plot of female ALS mice do not show any significant difference between the PBS injection group survival (black line) and the hiPSC-GRNPs transplantation group survival (green line) throughout the course of the study (n = 7 mice per group, P = 0.11363 stratified log-rank test).

Figure S2

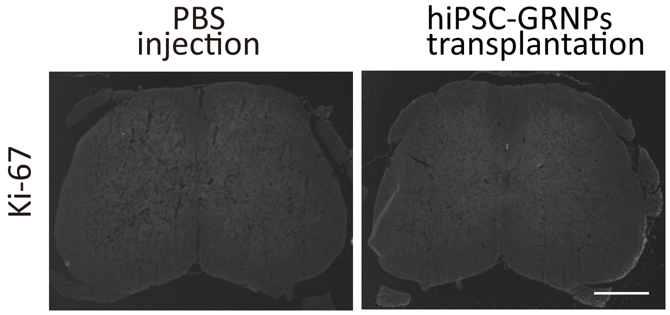

Figure S2, related to Figure 3. Histological evaluation for tumorigenicity

Tumorigenicity after transplantation was evaluated by immunostaining for Ki-67, a marker of proliferating cells. Tumor-like graft mass was not observed. No Ki67-positive grafts were found. Scale bar = 500 μm.

Figure S3

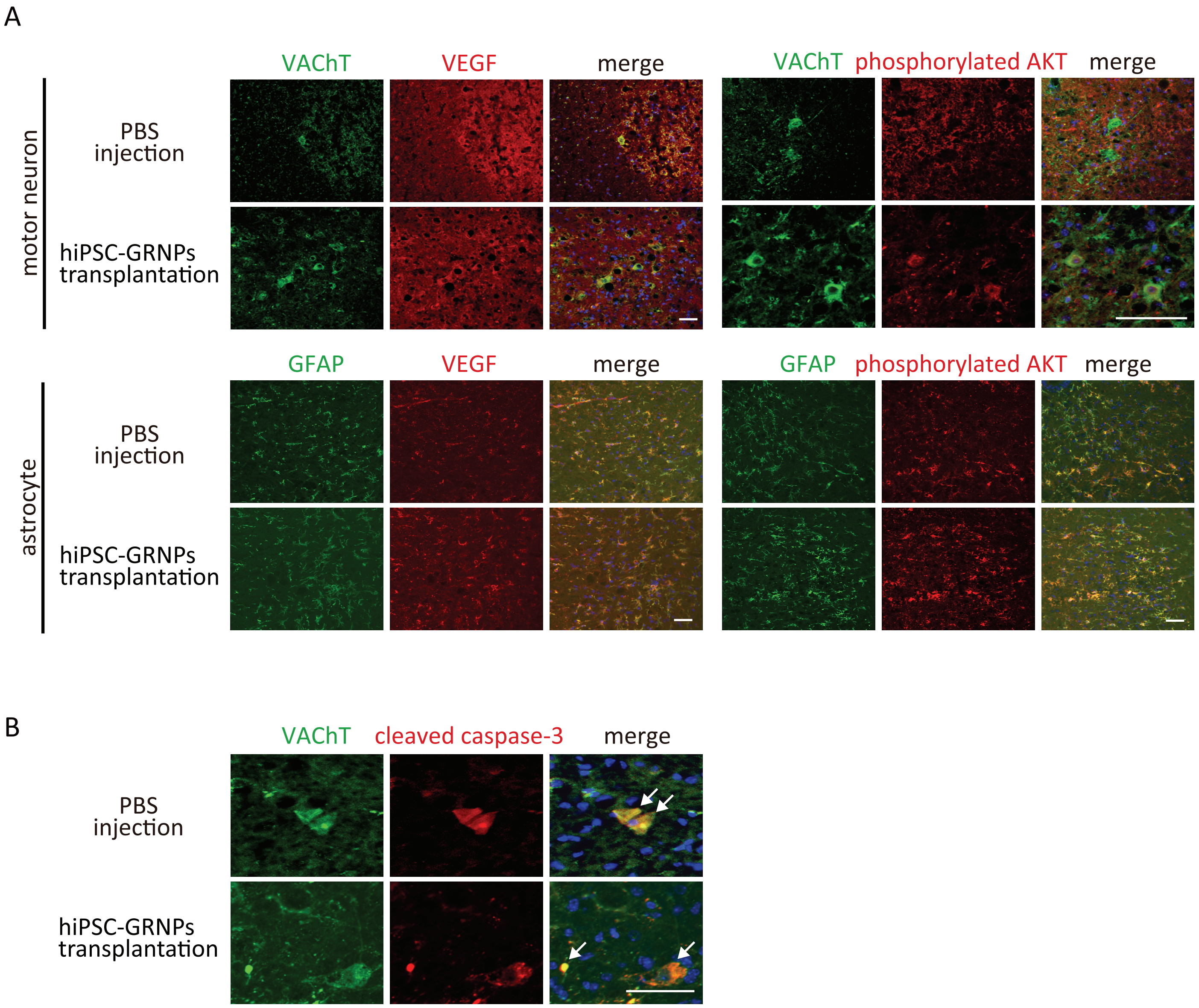
A
VAChT
VEGF
merge
VAChT
phosphorylated AKT
merge
motor neuron
PBS injection
hiPSC-GRNPs transplantation
GFAP
VEGF
merge
GFAP
phosphorylated AKT
merge
astrocyte
PBS injection
hiPSC-GRNPs transplantation
B
VAChT
cleaved caspase-3
merge
PBS injection
hiPSC-GRNPs transplantation

Figure S3, related to Figure 4. Histological evaluation of the effect of transplantation

(A) VEGF and phosphorylated AKT, and (B) cleaved caspase-3 were immunostained. VAChT and GFAP are motor neuron and astrocyte markers, respectively. Scale bars = 50 μm.

Table S1, related to Figure 2. Comparison with previous transplantation research with ALS model animals

study no.	graft type	host	transplantation site and condition	TP day	control	immunosupression	results and mechanistic insights	sex comparison	reference
1	human, NPC (22-week fetus) secreting human GDNF	rat	lumbar<br>12 x10^4 cells in 2 µl<br>by glass capillary	90-100	vehicle solution	cyclosporine A	no benefit on survival<br>increased ChAT+ soma size	not done	Klein SM et al. Hum Gene Ther. 2005 16(4):509-21.
2	human, NSC (8-week fetus)	rat	lumbar<br>5 x10^4 cells in 1 µl<br>by glass capillary	62	dead cells	FK-506	improved survival<br>upregulation of GDNF/BDNF	not done	Xu L et al. Transplantation. 2006 15;82(7):865-75.
3	mouse, NSC (6~8-week fetus)	mouse	lumbar<br>1 x10^4 cells in 1 µl<br>by glass capillary	70	vehicle solution	not used	improved survival<br>upregulation of VEGF/IGF1	not done	Corti S et al. Brain. 2007 130(5):1289-305.
4	mouse, olfactory bulb NSC	mouse	lumbar<br>500 neurospheres in 2 µl<br>by steel needle	70	vehicle solution	not used	improved survival<br>graft axons in ventral root	not done	Martin LJ et al. Neuropathol Exp Neurol. 2007 66(11):1002-18.
5	human, NPC (10~15-week fetus) secreting human GDNF	rat	lumbar<br>18 x10^4 cells in 2 µl<br>by glass capillary	70	vehicle solution	cyclosporine A	prevented motor neuron death	female only	Suzuki M et al. PLoS One. 2007 1;2(8):e689.
6	rat, GRPs (2-week fetus)	rat	cervical<br>15 ×10^4 cells in 2 µl<br>by 30G needle	90	vehicle solution or dead cells	cyclosporine A	improved survival<br>upregulation of GLT1<br>decreased microgliosis	not done	Lepore AC et al. Nat Neurosci. 2008 ;11(11):1294-301.
7	human, GRPs (17~24-week fetus)	mouse	cervical<br>5 x10^4 cells, 2 µl<br>by 30G needle	50-60	fibroblast	cyclosporine A	no benefit	not done	Lepore AC et al. PLoS One. 2011;6(10):e25968.
8	human, GRPs (17~24-week fetus)	mouse	cervical<br>5 x10^4 cells, 2 µl<br>by 30G needle	50-60	fibroblast	FK-506<br>rapamycin	no benefit	not done	Lepore AC et al. PLoS One. 2011;6(10):e25968.
9	human, GRPs (17~24-week fetus)	mouse	cervical<br>15 x10^4 cells, 2 µl<br>by 30G needle	50-60	fibroblast	FK-506<br>rapamycin	no benefit	not done	Lepore AC et al. PLoS One. 2011;6(10):e25968.
10	human, NSC (8-week fetus)	rat	cervical and lumbar<br>2 x10^4 cells in 1 µl<br>by 33G needle	62	dead cells	FK-506	improved survival	not done	Xu L et al. Neurosci Lett. 2011 2;494(3):222-6.
11	human, NSC (fetus)	rat	lumbar<br>1 x10^4 cells in 0.5 µl<br>by glass capillary	60-65	vehicle solution	FK-506<br>mycophenolate mofetil	transiently improved hind limb motor score<br>no benefit on survival	no significance in analyzing males and females separately	Hefferan MP et al. PLoS One. 2012;7(8):e42614.
12	human, iPSC derived NSC	mouse	into tail vein<br>100 x10^4 cells in 100 µl	90	vehicle solution	not used	improved survival<br>decreased microgliosis/astrogliosis	not done	Nizzardo M et al. Hum Mol Genet. 2014 15;23(2):342-54.
13	human, iPSC derived NSC	mouse	intrathecal<br>100 x10^4 cells in 5 µl	90	vehicle solution	not used	improved survival<br>decreased microgliosis/astrogliosis	not done	Nizzardo M et al. Hum Mol Genet. 2014 15;23(2):342-54.
14	human, iPSC derived GRNPs	mouse	lumbar<br>4 x10^4 cells in 0.5 µl<br>by 35G needle	90	vehicle solution	FK-506	improved survival<br>upregulation of VEGF/phosphorylated AKT	significant only in male	this study

Vehicle solution includes PBS, saline and culture medium.
Abbreviation: NPC, neural progenitor cells; NSC, neural stem cells; GRP, glial-restricted progenitors; GDNF, glial cell line derived neurotrophic factors; TP, transplantation

Table S2, related to Figure 4. Primer names and sequences used in species-specific qPCR

Primer name	Forward	Reverse	Target Species	Amplicon Position
**rt_VEGF_Hu022**	cattggagccttgccttg	atgattctgccctcctcctt	Human	Exon1/2
**rt_VEGF_Ms022**	actggaccctggctttactg	tctgctctccttctgtcgtg	Mouse	Exon1/2
**rt_NGF_Hu032**	tccggacccaataacagttt	catggacattacgctatgcac	Human	Exon2/3
**rt_NGF_Ms096**	tatactggccgcagtgaggt	ggacattgctatctgtgtacgg	Mouse	Exon2/3
**rt_NTF3_Hu018**	gcgacaacagagacgctaca	cacgtaatcctccatgagatacaa	Human	Exon3
**rt_NTF3_Ms091**	ggaggaaacgctatgcagaa	gtcacccacaggctctcact	Mouse	Exon3
**rt BDNF_Hu054**	cggaaggacctatgtttgct	tatttcagaacgcgcaactg	Human	Exon2
**rt_BDNF_Ms031**	gtggtgtaagccgcaaaga	aaccatagtaaggaaaaggatggtc	Mouse	Exon2
**rt_HGF_Hu056**	gaaggatcagatctggttttaatga	tgcatccataattaggtaaatcaatc	Human	Exon15/16
**rt_HGF_Ms033**	gcgcaagcagatcttaaaca	aagttatccaggattgcaggtc	Mouse	Exon15/16
**rt_GDNF_Hu006**	agctgccaacccagagaat	aaatgtattgcagttaagacacaacc	Human	Exon3
**rt_GDNF_Ms026**	cctcgaagagagaggaatcg	cgacctttccctctggaat	Mouse	Exon3

Supplemental Experimental Procedures

GFP-labeled iPSC generation

Human iPSC 201B7 was cultivated on an SNL feeder cell layer in primate embryonic stem cell medium (ReproCELL Inc., Japan) supplemented with 4 ng/ml basic FGF (Wako Pure Chemicals Industries, Ltd, Japan). The medium was changed every day. The *piggyBac* transposon vector (PB-EF1alpha-EiP) was constructed to express EGFP and puromycin resistance gene under control of the human EF1α promoter. We transfected 201B7 human iPS cells with PB-EF1alpha -EiP and *piggyBac* transposase expressing plasmids by using FuGENE HD (Promega) reagent on Matrigel (Becton Dickinson)-coated plates. Puromycin selection (1 μg/ml) was applied from 3 days after transduction, and was maintained for several passages to ensure stable and long-term expression of EGFP. After GFP-labeling and puromycin selection, 46XX karyotype was confirmed by Mitsubishi Chemical Medience Corporation.

Preparation of hiPSC-GRNPs for transplantation

hiPSCs were dissociated to single cells and quickly reaggregated in U-bottom 96-well plates (Greiner bio-one, Germany), pre-coated with 2% Pluronic F-127 (Sigma-Aldrich) in 100% ethanol, for suspension culture. Aggregations, EBs, were cultured in ‘DFK5% medium’ (DFK5%; DMEM/Ham’s

F12 (Gibco) supplemented with 5% KSR (Gibco), NEAA (Invitrogen, Japan), L-glutamine (Sigma-Aldrich), 0.1 μM 2-mercaptoethanol (Invitrogen)) with 2 μM dorsomorphin (Sigma-Aldrich) and 10 μM SB431542 (Cayman Chemical, U.S.A.) during the neural inductive stage (day 0 to 8). After the neural-induction stage, EBs were transferred onto Matrigel (Becton Dickinson)-coated 6-well culture plates and cultured in DFK5% supplemented with 1x N2 supplement (Invitrogen), 10 ng/ml human BMP4 (R&D, USA), and 10 ng/ml human LIF (R&D) during the patterning stage (day 8 to 28). Large numbers of neural precursor cells (NESTIN-positive) were observed to migrate from the edge of EBs. At day 28 *in vitro*, migrated neural precursor cells were harvested from the plates using Accutase (Innovative Cell Technologies, Inc.), centrifuged (200 g, 5 min), and resuspended in sterile PBS, without the addition of any neurotrophic factors at a concentration of 80,000 cells/μl. After completion of the transplantation, we evaluated the viability of hiPSC-GRNPs by trypan-blue exclusion assay. Viability was always more than 90%.

Transplantation

The mice were anesthetized with pentobarbital sodium (20 mg/kg; in room air, Kyoritsuseiyaku Corporation) and placed into a spinal clamp apparatus (Narishige, Japan). Muscle and connective tissue were dissected, through the lamina of the Th12/L1 vertebrae, which corresponded to the L3-L4 segment

of the spinal cord. Each mouse received two grafts (bilaterally at L3-L4) of 4 x 10^4 cells (in 0.5 μl PBS) into the ventral horn. Cells were delivered by 10-μl Gastight syringe (Hamilton, Sigma, Japan) with a 35-gauge/25-degree beveled needle (React Systems, Osaka, Japan). The needle tip was positioned at 150 μm laterally from the mid-line, lowered to a depth of 1,000 μm below the surface of the spinal cord and was held in place for 2 min both before and after cell injection. Cells were delivered under the control of a microsyringe pump controller, Nanojet (CHEMIX, Stafford, USA) at a rate of 0.1 μl/min. Control animals were injected with 0.5 μl of PBS solution per horn in a similar fashion as the cell-transplanted animals. Tg-G93A SOD1 mice were divided into hiPSC-GRNPs transplantation (n = 24 total: n = 17 males, n = 7 females) and PBS injection group (n = 24 total: n = 17 males, n = 7 females). The study was designed such that littermates were distributed equally among the transplanted and non-transplanted groups. Immunosupressant (FK506 3 mg/kg/day; Astellas, Tokyo, Japan) was administered orally to both PBS injection and hiPSC-GRNPs transplantation groups, from one week before the operation for the duration of the study. For axon count, two groups of animals (each consisting of n = 3 males) were sacrificed at 120 days of age and excluded from survival assessment.

Animal care

All animals were cared for and procedures performed in accordance with the Kyoto University

Animal Institutional Guidelines and were maintained in a specific pathogen-free environment. All animal experiments were approved by the CiRA Animal Experiment Committee (No. 24 and No. 27). The ALS model mouse line (B6.Cg-Tg(SOD1*G93A)1Gur/J (Tg-G93A SOD1 mouse)) was obtained from Jackson Laboratories and Oriental BioService. Animals were housed under a light:dark (12:12h) cycle and provided with food and water *ad libitum*.

Assessment of neurological function and disease progression

Motor function was evaluated by clinical grading system (Figure S1A) (Zhou et al., 2007) and scored by two experimenters blinded to the treatment groups. To determine disease end-stage in a reliable and ethical fashion, end-stage was defined by the inability of mice to right themselves within 30 seconds when placed on their sides.

Immunohistochemistry

Mice were anesthetized with pentobarbital and perfused transcardially with ice-cold phosphate buffer saline (PBS, pH 7.4) and subsequently with 4% paraformaldehyde in PBS. All mice were perfused under identical conditions by the same individual. Tissues were then cryoprotected in a 20% sucrose solution in PBS at 4°C until they sank. They were frozen in Tissue-Tek O.C.T. Compound (Sakura

Finetek, Japan). We confirmed the injection sites by stereo microscope (Leica, Japan) and cut the lumbar spinal cord at injection sites to freeze samples in Tissue-Tek O.C.T. Compound. The spinal cords were sliced into 10-μm transverse sections on a cryostat at −20°C, and every tenth section from the injection sites was mounted on slide glass to be stored at −80°C until use. Tissue sections were permeabilized in PBS containing 0.1% Triton X-100 for 15 min at room temperature, followed by rinsing with PBS. Nonspecific binding was blocked with PBS containing 10% donkey serum for 60 min at room temperature. Slices were incubated with primary antibodies overnight at 4°C, and then labeled with appropriate fluorescent-tagged secondary antibodies. DAPI (Life Technologies) was used to label nuclei. Fluorescence images were acquired on a BioRevo fluorescent microscope (Keyence, Osaka, Japan), LSM710 microscope (Carl Zeiss, Göttingen, Germany), or In Cell Analyzer 6000 (GE Healthcare, Japan). The following primary antibodies were used in immunocytochemistry or immunohistochemistry: MAP2, microtubule-associated protein 2 (1:2,000; Millipore, Japan), GFAP, glial fibrillary acidic protein (1:2,000; DAKO, Japan), GLT-1, glutamate transporter 1 (1:200; Abnova, USA), ALDH1L1, aldehyde dehydrogenase 1 (1:400; Abnova), NESTIN (1:2,000; Millipore), TUJ1 (1:4,000; Millipore), A2B5 (1:400; Millipore), CNPase (1:400; Cell Signaling Technology, Japan), VAChT (1:400; Chemicon, Japan), cleaved caspase-3 (1:400; Cell Signaling Technology), phosphorylated AKT (1:400; Cell Signaling Technology), GFP, green fluorescence protein (1:3,000; Invitrogen) and Iba1 (1:400; Wako

Pure Chemicals Industries, Ltd., Japan). For evaluating the positive count ratio of immunocytochemistry, we imaged the cells in the ventral horn parenchyma using automated microscopy, IN Cell Analyzer 2000 or 6000 (GE Healthcare, Japan), and counted the immunostained structural components by using IN cell developer toolbox software (GE Healthcare).

Morphological analysis of axons

Lumbar roots, postfixed in 4% paraformaldehyde/PBS, were frozen in Tissue-Tek O.C.T. Compound (Sakura Finetek) and transversely sectioned into 5-μm slices at −20°C and stored at −80°C until use. Sliced lumbar roots were stained with neurofilament-H antibody (1:2,000; Millipore). Entire roots were imaged with a BioREVO fluorescent microscope. Axonal diameters of L4 roots were measured by ImageJ software and grouped into 1-μm bins.

Human- or mouse-specific quantitative RT-PCR for spinal cord

Total RNA from the injection sites of mouse lumbar spinal cords was extracted by RNeasy plus kit (QIAGEN). Five-hundred nanograms of total RNA was reverse-transcribed into cDNA using RevaTra Ace with random primers (Toyobo, Osaka, Japan). To analyze separate gene expressions from graft or host origin, we designed human- or mouse-specific PCR primers specific for VEGF, vascular endothelial

growth factor; NGF, nerve growth factor; NT3, neurotrophin-3; BDNF, brain-derived neurotrophic factor; GDNF, glial cell-line derived neurotrophic factor; and HGF, hepatocyte growth factor (Table S2). One μL of the generated cDNA was used as template for each reaction in real-time quantitative PCR analysis, using SYBR Green II (TAKARA, Shiga, Japan) and StepONE plus (Life Science Technology). GAPDH was used as endogenous control gene to normalize target genes. To perform relative quantification, the comparative threshold (Ct) cycle method was used. The fold change in gene expression profile was referred to a PBS-injection group.

Western blot analysis

Tissues, removed from mice, were lysed in RIPA buffer (50 mM Tris-HCl buffer, pH 8.0, 150 mM NaCl, 1% NP-40, 0.5% deoxycholate, 0.1% SDS, and protease inhibitor cocktail (Roche Diagnostics)). The lysates (20 μg/lane) were subjected to SDS-PAGE and then electrically blotted to a polyvinylidene difluoride sheet. The sheet was soaked with an appropriate first antibody and subsequently with HRP-labeled anti-mouse IgG or anti-rabbit IgG (Bio-Rad). The following primary antibodies were used in western blot analysis: total AKT (1:3,000; Cell Signaling Technology), phosphorylated AKT (1:3,000; Cell Signaling Technology), VEGF (1:3,000; Abcam) and β-Actin (1:20,000; Sigma). The antigenic bands were visualized with ECL prime (GE Healthcare). The images were acquired on LAS 4000 (GE

Healthcare). The intensity of the protein band was analyzed using ImageQuant TL software (GE Healthcare).

Statistical analysis

We used JMP® 9 software for statistical calculation and drawing survival time graphs (SAS Institute Inc., Cary, NC, USA).

Supplemental reference

Zhou, C., Zhao, C.P., Zhang, C., Wu, G.Y., and Xiong, F. (2007). A method comparison in monitoring disease progression of G93A mouse model of ALS. Amyotroph Lateral Scler *8*, 366-372.
