# Supplementary material for: Focal Transplantation of Human iPSC-Derived Glial-Rich Neural Progenitors Improves Lifespan of ALS Mice
Source: Stem Cell Reports. 2014 Jun 26;3(2):242–9. doi: 10.1016/j.stemcr.2014.05.017 (PMC4175543; doi:10.1016/j.stemcr.2014.05.017)
Supplement: Document S2. Article plus Supplemental Information [file mmc2.pdf]

Stem Cell Reports
Report
OPEN ACCESS

Focal Transplantation of Human iPSC-Derived Glial-Rich Neural Progenitors Improves Lifespan of ALS Mice

Takayuki Kondo,[1,2,3] Misato Funayama,[1,3] Kayoko Tsukita,[1,3] Akitsu Hotta,[1,3,4,5] Akimasa Yasuda,[6] Satoshi Nori,[6] Shinjiro Kaneko,[6,7] Masaya Nakamura,[6] Ryosuke Takahashi,[2] Hideyuki Okano,[8] Shinya Yamanaka,[1,9] and Haruhisa Inoue[1,3,*]
[1]Center for iPS Cell Research and Application (CiRA), Kyoto University, Kyoto 606-8507, Japan
[2]Department of Neurology, Graduate School of Medicine, Kyoto University, Kyoto 606-8507, Japan
[3]CREST, JST, Saitama 332-0012, Japan
[4]PRESTO, JST, Saitama 332-0012, Japan
[5]iCeMS, Kyoto University, Kyoto 606-8507, Japan
[6]Department of Orthopedic Surgery, School of Medicine, Keio University, Tokyo 160-8582, Japan
[7]Department of Orthopaedic Surgery, National Hospital Organization, Murayama Medical Center, Tokyo 208-0011, Japan
[8]Department of Physiology, School of Medicine, Keio University, Tokyo 160-8582, Japan
[9]Gladstone Institute of Cardiovascular Disease, San Francisco, CA 94158, USA
*Correspondence: haruhisa@cira.kyoto-u.ac.jp
http://dx.doi.org/10.1016/j.stemcr.2014.05.017
This is an open access article under the CC BY license (http://creativecommons.org/licenses/by/3.0/).

SUMMARY

Transplantation of glial-rich neural progenitors has been demonstrated to attenuate motor neuron degeneration and disease progression in rodent models of mutant superoxide dismutase 1 (SOD1)-mediated amyotrophic lateral sclerosis (ALS). However, translation of these results into a clinical setting requires a renewable human cell source. Here, we derived glial-rich neural progenitors from human iPSCs and transplanted them into the lumbar spinal cord of ALS mouse models. The transplanted cells differentiated into astrocytes, and the treated mouse group showed prolonged lifespan. Our data suggest a potential therapeutic mechanism via activation of AKT signal. The results demonstrated the efficacy of cell therapy for ALS by the use of human iPSCs as cell source.

INTRODUCTION

Amyotrophic lateral sclerosis (ALS) is a disorder of motor neurons (MNs) that is characterized by their relatively rapid degeneration, resulting in progressive muscle weakness and respiratory failure (Bruijn et al., 2004). Approximately 90%–95% of ALS cases are sporadic in nature, with 20% of the remaining familial cases linked to various point mutations in the Cu/Zn superoxide dismutase 1 (SOD1) gene. Transgenic mice and rats carrying ALS-associated mutant human SOD1 genes (mSOD1) recapitulate many features of the human disease (Gurney et al., 1994).

Despite the relative selectivity of MN loss in ALS, studies in mSOD1 rodent and tissue culture models show non-neuronal (glial) cell involvement in the disease process (Boillée et al., 2006; Yamanaka et al., 2008). Astrocytes in particular are hypothesized to play a role in both mSOD1 and sporadic forms of ALS (Haidet-Phillips et al., 2011; Howland et al., 2002; Papadeas et al., 2011). Regardless of whether astrocyte dysfunction is a cause of the disease or a consequence of neuronal death, altered astrocyte physiology results in further susceptibility to MN loss (Boillée et al., 2006). Targeted enrichment of normal astrocytes in mSOD1 rat spinal cord via intraspinal transplantation of rodent glial-restricted progenitors promoted focal MN protection, delayed decline in respiratory function, and extended disease progression (Xu et al., 2011).

Various kinds of cells have been investigated for transplantation studies (Corti et al., 2004; Garbuzova-Davis et al., 2008; Iwanami et al., 2005; Piccini et al., 1999). Neuronal cells are probably the most relevant cell type for ALS treatment, but such cells suffer from a limited supply, ethical issues, and/or invasive harvest from human donors. On the other hand, human induced pluripotent stem cells (hiPSCs) can be obtained from a donor less invasively and can be expanded indefinitely in vitro. In this context, here we established a differentiation protocol of glial-rich neural progenitors (GRNPs) from hiPSCs and investigated the potential of hiPSC-derived glial-rich neural progenitors (hiPSC-GRNPs) as a cell source for intraspinal transplantation therapy of ALS.

RESULTS

Cell Resource Establishment for Transplantation

As a cell resource, we selected human iPSC line "201B7 clone," which had been previously evaluated as possessing low tumorigenicity after transplantation therapy (Kobayashi et al., 2012; Nori et al., 2011) To distinguish the transplanted cells from host cells, we introduced a *piggyBac* vector, which stably expresses GFP gene under the control of the ubiquitous EF1α promoter, into hiPSCs and observed continuous GFP fluorescence even after neural-lineage

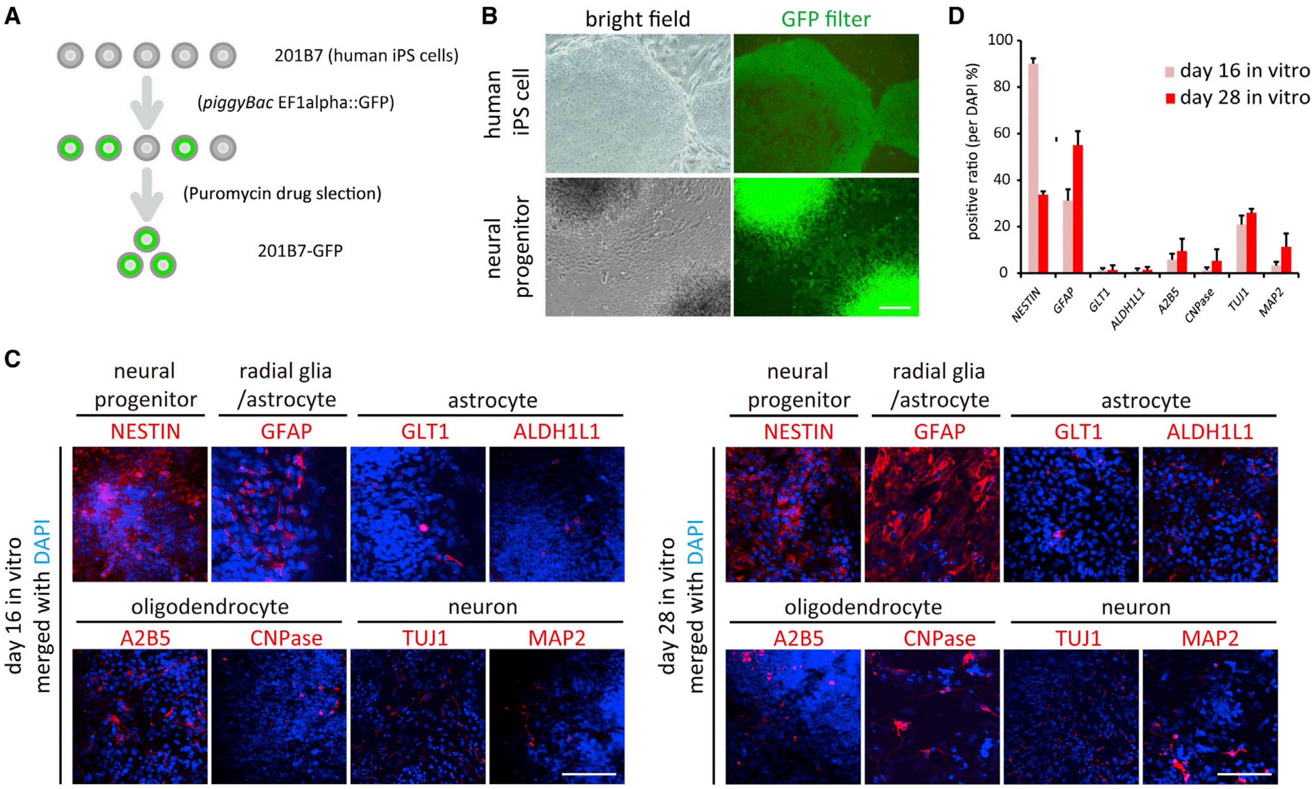

Figure 1. Human iPSCs Were Labeled with GFP, and They Differentiated into Neural Precursors
(A) hiPSCs were labeled with GFP by a *piggyBac* vector.
(B) GFP-labeled hiPSCs retained GFP signals after neural induction.
(C) hiPSC-derived neural precursors exhibited immunoreactivities for NESTIN (neural precursor marker), GFAP (astroglial or radial-glial marker), GLT1/ALDH1 (functional/mature astrocyte marker), A2B5/CNPasae (oligodendrocyte lineage marker), and TUJ1/MAP2 (neural lineage marker).
(D) Quantification of hiPSC differentiation in (C).
Data represent mean ± SD (n = 3 experiments). Scale bars, 200 μm.

differentiation (Figures 1A and 1B). We differentiated GFP-labeled hiPSCs into neural stem cells by the serum-free floating culture of embryoid bodies-like aggregates method with SMAD-pathway inhibition (Kondo et al., 2013). Neural stem cells were efficiently differentiated into hiPSC-GRNPs by stimulation of the LIF/BMP signaling. This protocol provided highly enriched neural precursors, 68.4% ± 7.2% positive for NESTIN and 54.9% ± 6.1% positive for GFAP (Figures 1C and 1D). At day 16 in vitro, most of the differentiated grafts were positive for NESTIN or GFAP. At this very early stage, $GFAP^+$ cells include either radial glia, a subtype of developmental neural progenitors with a neuron-like spine, or immature astrocytes (Liour and Yu, 2003). At day 28 in vitro, $NESTIN^+$ neural progenitors differentiated into $TUJ1^+$ neurons, $A2B5^+$ oligoprogenitors, and $GFAP^+$ astrocytes. The differentiation method used in the present work could augment the $GFAP^+$ glial population and attenuate TUJ1 neural differentiation, as compared with our previous method (Kondo et al., 2013). However, $GFAP^+$ astrocytes were not positive for GLT1 or ALDH1L1, which were thought to be functionally mature astrocytes before transplantation.

hiPSC-GRNPs Transplantation Improved Motor Function and Survival in ALS Model Mice

All animal experiments were approved by the CiRA Animal Experiment Committee (nos. 24 and 27). We transplanted 40,000 hiPSC-derived GRNPs each into bilateral lumbar spinal cords of transgenic SOD1-G93A mice. Transplantation operations were performed after onset of ALS phenotype, at 90 days of age to mimic the clinical situation (Figure 2A). Littermates of transplanted mice received only a vehicle (PBS) injection and were used as control group. We designed the study so that siblings were distributed equally in the control (n = 24, male:female = 17:7) and transplanted (n = 24, male:female = 17:7) groups. By using a 35 gauge needle and a relatively small injection volume, we could avoid motor disturbance at 24 hr after the surgical

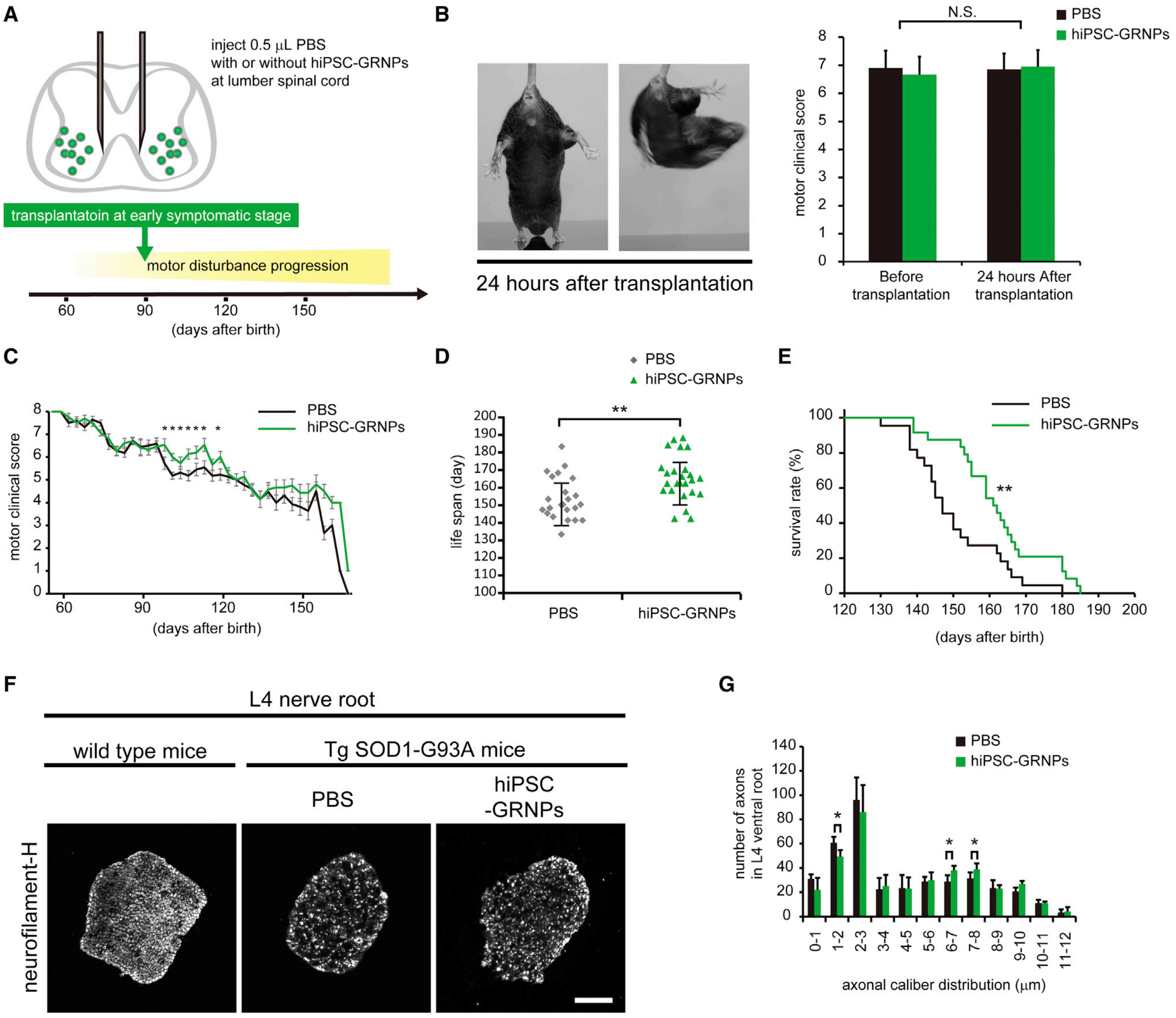

Figure 2. hiPSC-GRNPs Transplantation Improved Motor Score and Survival in ALS Model Mice

(A) Transplantation schedule and schema of spinal cord injection site. Transplantation was performed after disease onset.

(B) Mice presented with no side effects after transplantation and made vigorous twisting movements with hind-limb extension, as shown by representative photos at tail suspension. Both groups showed no change in motor clinical score at 24 hr after surgical insult.

(C) Clinical motor scoring change by sequential evaluation showed significant difference from 100 to 120 days after birth (*$p < 0.05$). Data represent mean ± SEM (n = 21 mice per group).

(D) Lifespan was prolonged in the hiPSC-GRNPs transplantation group (162.2 ± 12.8 days) compared to the control group (150.4 ± 12.1 days) (**$p < 0.01$). Data represent mean ± SD (n = 21 mice per group).

(E) Survival (Kaplan-Meier plot) analysis shows a significant difference between the PBS injection group survival (black line) and the hiPSC-GRNPs transplantation group survival (green line) throughout the course of the study (n = 21 mice per group, p = 0.00691 stratified log-rank test), suggesting that the hiPSC-GRNPs transplantation group had better survival (**$p < 0.01$).

(F) The number of axons in L4 ventral nerve root was counted to estimate surviving motor neurons at the middle stage of disease progression. At 120 days after birth, transverse sections of ventral nerve roots were stained with an anti-neurofilament-H antibody. Compared to littermates without transgene, the number of axons in Tg SOD1-G93A mice was decreased. Each axon caliber was measured and classified according to size.

(G) Cumulative axon caliber distribution at L4 ventral root at 120 days after birth of both groups. Two-way ANOVA with repeated-measures was used to study the effect of transplantation (transplanted and nontransplanted mice) on axonal caliber distribution. Pairwise

(legend continued on next page)

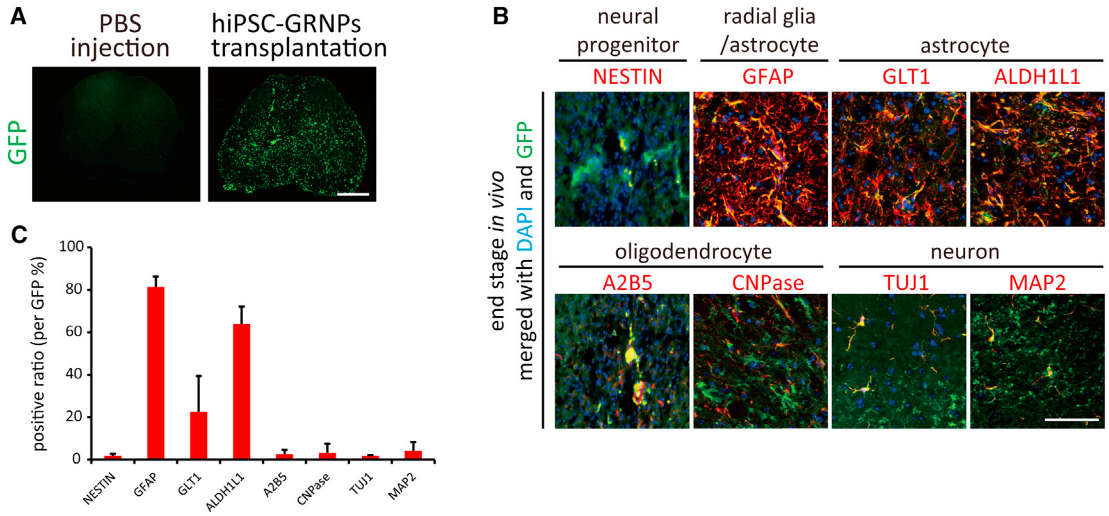

Figure 3. Transplanted Cells Differentiated Mainly into Astrocytes

(A) Transplanted GFP-labeled grafts (green) survived in the spinal cord. Scale bars, 500 μm.

(B) Most of the GFP-labeled grafts in ventral horn parenchyma differentiated into mature astrocytes, which were positive for astrocyte markers (red), including GFAP, ALDH1L1, and GLT1, in vivo. A relatively small population of grafts also differentiated into CNPase$^+$ oligodendrocytes or MAP2$^+$ neurons. A limited number of grafts remained as NESTIN$^+$ neural progenitors. Scale bar, 50 μm.

(C) Quantification of positive ratio of cell type markers in (B). A small number of GFP-labeled grafts were also positive for A2B5, an oligoprogenitor marker, MAP2, a neuronal marker, or Nestin, a neural precursor marker, in vivo. The total number of GFP-positive cells, counted to calculate the positive ratio, was 113.3 ± 14.7 for NESTIN, 75.9 ± 11.8 for GFAP, 68.7 ± 5.5 for GLT1, 76.9 ± 9.4 for ALDH1L1, 67.3 ± 13.0 for A2B5, 69.3 ± 19.2 for CNPase, 70 ± 13.6 for TUJ1, and 66.7 ± 20.2 for MAP2, respectively. Data represent mean ± SD (n = 3 mice per group). See also Figure S2.

insult (Figure 2B), evaluated by clinical grading system (Table S1 available online).

At 10–40 days after the procedure, we observed improvement in clinical motor score in the hiPSC-GRNPs transplantation group (Figure 2C). Surviving lifespan was extended by 7.8% in the hiPSC-GRNPs transplantation group (n = 21, male:female = 14:7, 162.2 ± 12.8 days) compared to the control group (n = 21, male:female = 14:7, 150.4 ± 12.1 days) (Figures 2D and 2E). When the effect of transplantation was evaluated separately in male and female mice, a greater survival improvement was noted in males than in females (Figure S1). Survival lifespan was significantly expanded only in males (145.3 ± 9.8 days for control, 158.5 ± 11.2 days for the hiPSC-GRNPs transplantation group, extended by 9.1%), not in females (160.5 ± 9.5 days for control, 169.7 ± 12.6 days for the hiPSC-GRNPs transplantation group, extended by 5.7%). To evaluate motor neuron degeneration at the symptomatic phase, three male mice from each group were sacrificed at 120 days of age. The number of 6–8 μm large-caliber fibers was increased in the hiPSC-GRNPs transplantation group (Figures 2F and 2G). We could not detect GFP signals, derived from transplanted hiPSC-GRNPs, in nerve root slices.

Transplanted hiPSC-GRNPs Differentiated into Astrocytes in Spinal Cord of ALS Model Mice without Tumorigenic Formation

We continued to evaluate clinical motor function (Table S1) and defined clinical grade 0 as end stage. At the end stage of disease progression, around 140–170 days after birth, the animals were sacrificed and histological analysis was performed to investigate the state of the engraftment. Transplanted hiPSC-GRNP-derived cells, which are positive for GFP signals, continued to survive in the lumbar spinal cord of ALS model mice (Figure 3A). Engraftment could be observed at least 5 mm away from the injection site. We assessed some cell subtype markers by immunostaining,

comparisons were made using Bonferroni adjustment (*$p < 0.05$). The number of 1–2 μm caliber axons was significantly decreased and the number of 6–8 μm caliber axons was significantly increased in the hiPSC-GRNPs transplantation group. Data represent mean ± SD (n = 3 mice per group).

Scale bars, 100 μm. hiPSCs, human induced pluripotent stem cells; hiPSC-GRNPs, hiPSC-derived glial-rich neural progenitors. See also Figure S1 and Table S1.

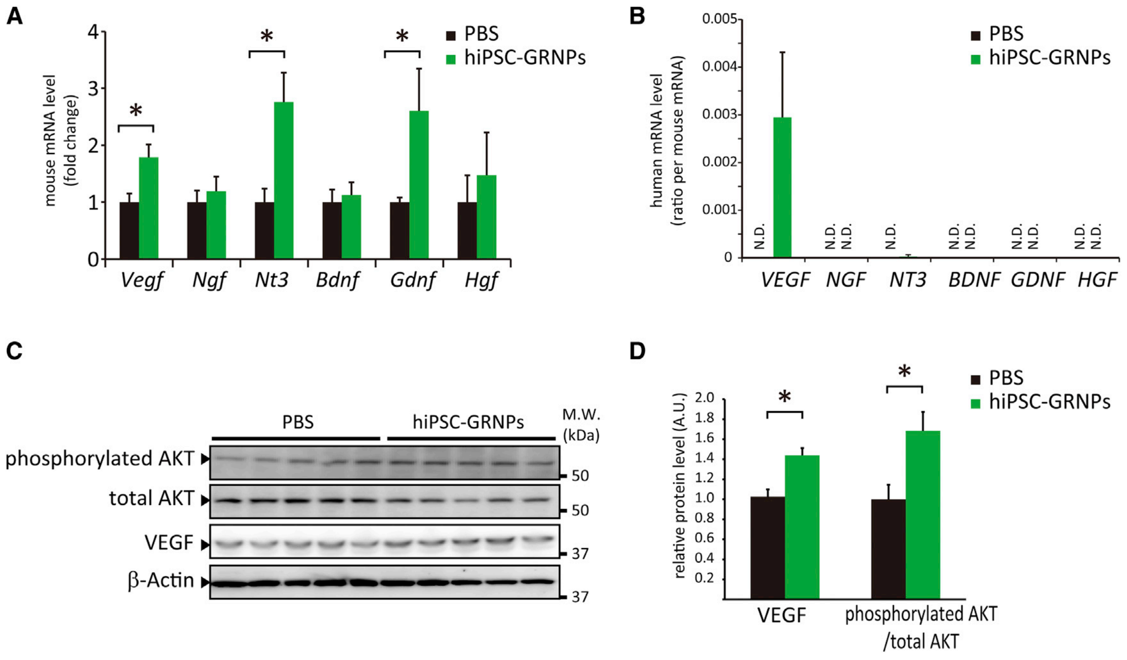

Figure 4. hiPSC-GRNPs Transplantation Increased Neurotrophic Factors and Activated AKT Signal

(A) Gene expressions of neurotrophic factors, including *Vegf*, *Ngf*, *Nt3*, *Bdnf*, *Gdnf*, and *Hgf* were quantitatively analyzed using mouse-specific primers. The levels of *Vegf*, *Nt3*, and *Gdnf* were significantly increased in the hiPSC-GRNPs transplantation group (*$p < 0.05$). Data represent mean ± SD (n = 3 mice per group).

(B) Gene expressions of neurotrophic factors, including *VEGF*, *NGF*, *NT3*, *BDNF*, *GDNF*, and *HGF* were quantitatively analyzed using human-specific primers and the human-origin/mouse-origin ratio was calculated. Data represent mean ± SD (n = 3 mice per group).

(C) Western blot analysis of phosphorylated AKT, total AKT level, and VEGF in lumbar spinal cord in the PBS injection group and hiPSC-GRNPs transplantation group.

(D) Densitometric analysis of (C). Measured values of proteins were normalized by that of β-actin. The levels of VEGF and AKT phosphorylation were significantly increased in the hiPSC-GRNPs transplantation group (*$p < 0.05$).

Data represent mean ± SD (n = 5 mice per group). N.D., not detectable. See also Figure S3 and Table S2.

including GFAP/GLT1/ALDH1L1 for astrocytes (Figure 3B), A2B5 for oligodendroglial progenitors, and NESTIN for neural progenitors (Figure 3C). Around 60%–80% of the cells at the grafts were double-positive for GFP fluorescence and GFAP marker, suggesting that transplanted hiPSC-GRNPs had differentiated into astrocytes (Figure 3C). Although we hardly observed the immunoreactivity of functional/mature astrocyte markers including GLT1 and ALDH1L1 in vitro before transplantation, we did observe it after transplantation. The rate of neurons or oligodendrocytes was low (Figure 3C). Only a small population of grafts retained positive staining for NESTIN, that is, remaining in the neural progenitor stage of differentiation (Figure 3C).

It is important to note that, during our observation period (up to 3 months posttransplantation), the injection sites showed no signs of tumor formation. Gross pathological examinations of other organs outside the CNS did not reveal any heterotopic engraftment.

Transplanted hiPSC-GRNPs Upregulated Neurotrophic Factors and Activated Cell Survival Signal

We investigated the expression level of neurotrophic factors in lumbar spinal cord. We designed mouse- or human-specific primers to evaluate host- or graft-derived mRNA separately (Table S2). Quantitative RT-PCR revealed that upregulated expressions were observed in mouse (host)-originated *Vegf*, *Nt3*, and *Gdnf*, but not in *Ngf*, *Bdnf*, or *Hgf* (Figure 4A). However, human (graft)-originated expression, equal to hiPSC-GRNPs origin, was observed only in *VEGF* (Figure 4B). Western blot analysis demonstrated a significant increase in VEGF level in the hiPSC-GRNPs transplantation group (Figures 4C and 4D). Furthermore, hiPSC-GRNPs transplantation increased phosphorylated AKT and activated AKT signaling, which is downstream from the VEGF signal and is important for cell survival in ALS (Lunn et al., 2009) (Figures 4C and 4D).

DISCUSSION

Here, we describe that transplantation of human iPSC-derived GRNPs produced astrocytes in vivo and prolonged the survival period of mSOD1 mice. We used hiPSC-GRNPs for testing the efficacy in mSOD1 mice, because replacement therapy using astrocytes from rodent glial-restricted progenitors in the cervical spinal cord of ALS rodent models is already well established (Xu et al., 2011).

We showed that glial cells represent a potential target of ALS therapy. However, we observed transient improvement of lower limb function, as shown in Figure 2C, and a similar previous study failed to show improvement in the rescue of clinical manifestations and neuronal survival by transplantation of human-derived glial-restricted progenitor cells from 17- to 24-week fetus into SOD1 transgenic mouse spinal cord despite the survival and proliferation of exogenous astrocytes (Lepore et al., 2011). Although the transient improvement in our study might have stemmed from neuroprotective effects of the transplanted cells only in the lumbar region, with a possible broader effect of neurotrophic factors on other regions or behavioral alteration for food intake, as previously discussed (Table S1), we comprehensively compared the two studies as well as others regarding lumbar transplantation in terms of a number of aspects (Table S1), speculating that there were differences in graft type, transplantation condition, and/or transplantation timing. Regarding the timing of transplantation, the survival improvement in our study might have resulted in attenuation of the glial contribution to the disease pathogenesis at an early symptomatic stage (Boillée et al., 2006; Yamanaka et al., 2008). Regarding the cell injection site, instead of the cervical cord, we injected the cells into the lumbar spinal cords of ALS model mice, which resulted in improved clinical scores of lower limbs. These data supported the possibility of targeting not only the cervical cord but also the lumbar spinal cord in ALS clinical trials, depending on the symptoms to be treated. Following previous transplantation research (Table S1), we selected PBS, which is a vehicle solution for grafts, as control agent of transplantation. Dead cells or fibroblasts can be appropriate control agents but may also possibly secrete various factors. Furthermore, previously an extensive study showed that, as a control agent, there was no significant difference among vehicle solution, dead cells, and fibroblasts (Lepore et al., 2008).

iPSCs were previously reported to induce T cell-dependent immune response by direct transplantation of undifferentiated cells into syngeneic mice. However, a more detailed investigation proved that autologous transplantation of terminally differentiated cells derived from iPSCs or embryonic stem cells elicits only negligible immunogenicity (Araki et al., 2013; Okano et al., 2013). In our study, we did not observe excess inflammatory responses around transplanted cells under treatment of low-dose immunosuppression, suggesting that even if transplanted cells were not autologous, we could control the immune responses of the recipients by immunosuppressant treatment.

The increase in the levels of neurotrophic factors had been commonly observed in transplantation therapy of ALS models (Nizzardo et al., 2014; Teng et al., 2012). We observed that the transplanted hiPSC-GRNPs produced VEGF, and expressions of endogenous VEGF and other neurotrophic factors in the host mice were upregulated. A previous study showed that VEGF retrograde delivery with lentiviral vector could prolong the survival of ALS model mice by 30% (Azzouz et al., 2004) and that activated AKT signaling, which is downstream of VEGF, is important for cell survival in ALS (Lunn et al., 2009). Similarly in this study, transplanted hiPSC-GRNPs increased the VEGF level and prolonged the survival of mSOD1 mice. We could observe positive immunostaining for VEGF and phosphorylated AKT in both remaining motor neurons and astrocytes. However, we could not observe any morphological difference in motor neurons between control and transplanted groups at end-stage. We also speculated that, as shown in previous studies (Howland et al., 2002), transplanted hiPSC-GRNPs differentiated into astrocytes expressing glutamate transporter 1 (GLT1) might restore glutamate homeostasis in our study.

In our study, the males in both the control and hiPSC-GRNPs transplantation groups died long before the females, and this result is consistent with the previous reports of ALS model mice (Cervetto et al., 2013; Choi et al., 2008). However, improvement in male mice was greater than in females (Figure S1) Interestingly, a similar gender-dependent difference of therapeutic efficacy was reported in ALS model mice (Cervetto et al., 2013; Li et al., 2012). The epidemiological studies of sporadic ALS have shown that both incidence and prevalence of ALS are greater in men than in women and onset of the disease is also earlier for men than it is for women (McCombe and Henderson, 2010). Sex steroids are suggested to be involved in the gender difference in ALS, but the direct importance of estrogen is still controversial (Choi et al., 2008; Li et al., 2012). Moreover, male neural cells are reported to be more vulnerable to oxidative stress, induced by mutant SOD1 overexpression, than female neural cells (Li et al., 2012). In our study, transplanted cells were mainly differentiated into GFAP-positive astrocytes and upregulated VEGF. Furthermore, astrocytes can play neuroprotective roles from oxidative stress via VEGF (Chu et al., 2010). These findings suggest that hiPSC-GRNPs transplantation may ameliorate male-specific vulnerability to oxidative

stress and improve the survival lifespan of male mice. Further analysis would be necessary to elucidate VEGF-associated mechanisms in transplantation therapy.

In regard to safety, the potential tumorigenicity of grafts is a predominant concern. We used the human iPSC line "201B7," which was previously reported to be safe from the viewpoint of tumorigenesis (Kobayashi et al., 2012). Furthermore, we found no signs of tumor formation or Ki67-positive grafts (Figure S2). However, a very small proportion of grafts remained positive for the neural progenitor marker NESTIN at 3 months posttransplantation. We cannot exclude the risk of tumor formation from the remaining NESTIN-positive NPCs. It is important to evaluate tumorigenicity by longer-term observations for future clinical trials.

We tested the potential of cell therapy after onset of the disease in ALS model mice, because most human cases of ALS are sporadic and any treatment would be initiated after onset. Our study showed a modest lifespan prolongation compared to previous studies testing cell therapy before disease onset in ALS model mice. Future studies of transplantations, such as combinations with MN engraftment, will be required to accelerate ALS treatment toward restoration of MN function and ultimately the complete cure of ALS.

EXPERIMENTAL PROCEDURES

Preparation of hiPSC-GRNPs for Transplantation

We differentiated hiPSCs into neural lineage cells using a previously described differentiation protocol (Kondo et al., 2013), under the condition of additional 10 ng/ml human BMP4 (R&D Systems) and 10 ng/ml human LIF (R&D) during the patterning stage (days 8–28).

Transplantation

We transplanted 40,000 hiPSC-GRNPs into bilateral lumbar spinal cords of 90-day-old Tg SOD1-G93A mice. Each mouse received two grafts (bilaterally at L3-L4) of 4×10^4 cells (in 0.5 μl PBS) into the ventral horn.

Statistical Analysis

The Mann-Whitney test was used for the analysis of two populations of means, and p values <0.05 were considered significant. Repeated-measures two-way ANOVA, followed by the Tukey-Kramer test, was used for clinical motor scoring analysis. The Kaplan-Meier plot was used to evaluate survival time, and the log-rank test was applied to compare cumulative curves.

SUPPLEMENTAL INFORMATION

Supplemental Information includes Supplemental Experimental Procedures, three figures, and two tables and can be found with this article online at http://dx.doi.org/10.1016/j.stemcr.2014.05.017.

AUTHOR CONTRIBUTIONS

H.I. conceived the project; T.K. and H.I. designed the experiments; T.K. M.F., K.T., A.Y., and S.N. performed the experiments; T.K., M.N., H.O., and H.I. analyzed the data; A.H. and S.Y. contributed reagents, materials, and analysis tools; A.Y., S.N., S.K., M.N., R.T., and H.O. provided critical reading and scientific discussions; T.K., A.H., H.O., and H.I. wrote the paper.

ACKNOWLEDGMENTS

We would like to express our sincere gratitude to all of our coworkers and collaborators; to Yoshiko Karatsu for technical assistance; to Yasuhiro Watanabe for technical advice; and to Katsura Noda and Kazumi Murai for their valuable administrative support. We thank Astellas Pharma Inc. for FK506. This study was supported in part by a grant from the Funding Program of World-Leading Innovative R&D on Science and Technology (FIRST Program) of the Japan Society for the Promotion of Science (S.Y.), from the Leading Project of MEXT (S.Y. and H.I.), from CREST (H.I.), and from a Grant-in-Aid for Scientific Research on Innovative Area "Foundation of Synapse and Neurocircuit Pathology" (22110007) from the Ministry of Education, Culture, Sports, Science and Technology of Japan (H.I.). S.Y. is a member without salary of the scientific advisory board of iPS Academia Japan. H.O. is a paid scientific consultant to San Bio, Inc., Eisai Co., Ltd., and Daiichi Sankyo Co., Ltd.

Received: December 9, 2013
Revised: May 22, 2014
Accepted: May 23, 2014
Published: June 26, 2014

REFERENCES

Araki, R., Uda, M., Hoki, Y., Sunayama, M., Nakamura, M., Ando, S., Sugiura, M., Ideno, H., Shimada, A., Nifuji, A., and Abe, M. (2013). Negligible immunogenicity of terminally differentiated cells derived from induced pluripotent or embryonic stem cells. Nature *494*, 100–104.

Azzouz, M., Ralph, G.S., Storkebaum, E., Walmsley, L.E., Mitrophanous, K.A., Kingsman, S.M., Carmeliet, P., and Mazarakis, N.D. (2004). VEGF delivery with retrogradely transported lentivector prolongs survival in a mouse ALS model. Nature *429*, 413–417.

Boillée, S., Vande Velde, C., and Cleveland, D.W. (2006). ALS: a disease of motor neurons and their nonneuronal neighbors. Neuron *52*, 39–59.

Bruijn, L.I., Miller, T.M., and Cleveland, D.W. (2004). Unraveling the mechanisms involved in motor neuron degeneration in ALS. Annu. Rev. Neurosci. *27*, 723–749.

Cervetto, C., Frattaroli, D., Maura, G., and Marcoli, M. (2013). Motor neuron dysfunction in a mouse model of ALS: gender-dependent effect of P2X7 antagonism. Toxicology *311*, 69–77.

Choi, C.I., Lee, Y.D., Gwag, B.J., Cho, S.I., Kim, S.S., and Suh-Kim, H. (2008). Effects of estrogen on lifespan and motor functions in female hSOD1 G93A transgenic mice. J. Neurol. Sci. *268*, 40–47.

Chu, P.W., Beart, P.M., and Jones, N.M. (2010). Preconditioning protects against oxidative injury involving hypoxia-inducible factor-1 and vascular endothelial growth factor in cultured astrocytes. Eur. J. Pharmacol. *633*, 24–32.

Corti, S., Locatelli, F., Donadoni, C., Guglieri, M., Papadimitriou, D., Strazzer, S., Del Bo, R., and Comi, G.P. (2004). Wild-type bone marrow cells ameliorate the phenotype of SOD1-G93A ALS mice and contribute to CNS, heart and skeletal muscle tissues. Brain *127*, 2518–2532.

Garbuzova-Davis, S., Sanberg, C.D., Kuzmin-Nichols, N., Willing, A.E., Gemma, C., Bickford, P.C., Miller, C., Rossi, R., and Sanberg, P.R. (2008). Human umbilical cord blood treatment in a mouse model of ALS: optimization of cell dose. PLoS ONE *3*, e2494.

Gurney, M.E., Pu, H., Chiu, A.Y., Dal Canto, M.C., Polchow, C.Y., Alexander, D.D., Caliendo, J., Hentati, A., Kwon, Y.W., Deng, H.X., et al. (1994). Motor neuron degeneration in mice that express a human Cu,Zn superoxide dismutase mutation. Science *264*, 1772–1775.

Haidet-Phillips, A.M., Hester, M.E., Miranda, C.J., Meyer, K., Braun, L., Frakes, A., Song, S., Likhite, S., Murtha, M.J., Foust, K.D., et al. (2011). Astrocytes from familial and sporadic ALS patients are toxic to motor neurons. Nat. Biotechnol. *29*, 824–828.

Howland, D.S., Liu, J., She, Y., Goad, B., Maragakis, N.J., Kim, B., Erickson, J., Kulik, J., DeVito, L., Psaltis, G., et al. (2002). Focal loss of the glutamate transporter EAAT2 in a transgenic rat model of SOD1 mutant-mediated amyotrophic lateral sclerosis (ALS). Proc. Natl. Acad. Sci. USA *99*, 1604–1609.

Iwanami, A., Kaneko, S., Nakamura, M., Kanemura, Y., Mori, H., Kobayashi, S., Yamasaki, M., Momoshima, S., Ishii, H., Ando, K., et al. (2005). Transplantation of human neural stem cells for spinal cord injury in primates. J. Neurosci. Res. *80*, 182–190.

Kobayashi, Y., Okada, Y., Itakura, G., Iwai, H., Nishimura, S., Yasuda, A., Nori, S., Hikishima, K., Konomi, T., Fujiyoshi, K., et al. (2012). Pre-evaluated safe human iPSC-derived neural stem cells promote functional recovery after spinal cord injury in common marmoset without tumorigenicity. PLoS ONE *7*, e52787.

Kondo, T., Asai, M., Tsukita, K., Kutoku, Y., Ohsawa, Y., Sunada, Y., Imamura, K., Egawa, N., Yahata, N., Okita, K., et al. (2013). Modeling Alzheimer's disease with iPSCs reveals stress phenotypes associated with intracellular Aβ and differential drug responsiveness. Cell Stem Cell *12*, 487–496.

Lepore, A.C., Rauck, B., Dejea, C., Pardo, A.C., Rao, M.S., Rothstein, J.D., and Maragakis, N.J. (2008). Focal transplantation-based astrocyte replacement is neuroprotective in a model of motor neuron disease. Nat. Neurosci. *11*, 1294–1301.

Lepore, A.C., O'Donnell, J., Kim, A.S., Williams, T., Tuteja, A., Rao, M.S., Kelley, L.L., Campanelli, J.T., and Maragakis, N.J. (2011). Human glial-restricted progenitor transplantation into cervical spinal cord of the SOD1 mouse model of ALS. PLoS ONE *6*, e25968.

Li, R., Strykowski, R., Meyer, M., Mulcrone, P., Krakora, D., and Suzuki, M. (2012). Male-specific differences in proliferation, neurogenesis, and sensitivity to oxidative stress in neural progenitor cells derived from a rat model of ALS. PLoS ONE *7*, e48581.

Liour, S.S., and Yu, R.K. (2003). Differentiation of radial glia-like cells from embryonic stem cells. Glia *42*, 109–117.

Lunn, J.S., Sakowski, S.A., Kim, B., Rosenberg, A.A., and Feldman, E.L. (2009). Vascular endothelial growth factor prevents G93A-SOD1-induced motor neuron degeneration. Dev. Neurobiol. *69*, 871–884.

McCombe, P.A., and Henderson, R.D. (2010). Effects of gender in amyotrophic lateral sclerosis. Gend. Med. *7*, 557–570.

Nizzardo, M., Simone, C., Rizzo, F., Ruggieri, M., Salani, S., Riboldi, G., Faravelli, I., Zanetta, C., Bresolin, N., Comi, G.P., and Corti, S. (2014). Minimally invasive transplantation of iPSC-derived ALDH-hiSSCloVLA4+ neural stem cells effectively improves the phenotype of an amyotrophic lateral sclerosis model. Hum. Mol. Genet. *23*, 342–354.

Nori, S., Okada, Y., Yasuda, A., Tsuji, O., Takahashi, Y., Kobayashi, Y., Fujiyoshi, K., Koike, M., Uchiyama, Y., Ikeda, E., et al. (2011). Grafted human-induced pluripotent stem-cell-derived neurospheres promote motor functional recovery after spinal cord injury in mice. Proc. Natl. Acad. Sci. USA *108*, 16825–16830.

Okano, H., Nakamura, M., Yoshida, K., Okada, Y., Tsuji, O., Nori, S., Ikeda, E., Yamanaka, S., and Miura, K. (2013). Steps toward safe cell therapy using induced pluripotent stem cells. Circ. Res. *112*, 523–533.

Papadeas, S.T., Kraig, S.E., O'Banion, C., Lepore, A.C., and Maragakis, N.J. (2011). Astrocytes carrying the superoxide dismutase 1 (SOD1G93A) mutation induce wild-type motor neuron degeneration in vivo. Proc. Natl. Acad. Sci. USA *108*, 17803–17808.

Piccini, P., Brooks, D.J., Björklund, A., Gunn, R.N., Grasby, P.M., Rimoldi, O., Brundin, P., Hagell, P., Rehncrona, S., Widner, H., and Lindvall, O. (1999). Dopamine release from nigral transplants visualized in vivo in a Parkinson's patient. Nat. Neurosci. *2*, 1137–1140.

Teng, Y.D., Benn, S.C., Kalkanis, S.N., Shefner, J.M., Onario, R.C., Cheng, B., Lachyankar, M.B., Marconi, M., Li, J., Yu, D., et al. (2012). Multimodal actions of neural stem cells in a mouse model of ALS: a meta-analysis. Sci. Transl. Med. *4*, 165ra164.

Xu, L., Shen, P., Hazel, T., Johe, K., and Koliatsos, V.E. (2011). Dual transplantation of human neural stem cells into cervical and lumbar cord ameliorates motor neuron disease in SOD1 transgenic rats. Neurosci. Lett. *494*, 222–226.

Yamanaka, K., Chun, S.J., Boillee, S., Fujimori-Tonou, N., Yamashita, H., Gutmann, D.H., Takahashi, R., Misawa, H., and Cleveland, D.W. (2008). Astrocytes as determinants of disease progression in inherited amyotrophic lateral sclerosis. Nat. Neurosci. *11*, 251–253.

Stem Cell Reports, Volume *3*

Supplemental Information

Focal Transplantation of Human iPSC-Derived Glial-Rich Neural Progenitors Improves Lifespan of ALS Mice

Takayuki Kondo, Misato Funayama, Kayoko Tsukita, Akitsu Hotta, Akimasa Yasuda, Satoshi Nori, Shinjiro Kaneko, Masaya Nakamura, Ryosuke Takahashi, Hideyuki Okano, Shinya Yamanaka, and Haruhisa Inoue

Figure S1

A

Grade	Clinical manifestation
8	Normal functions, no sign of disease onset
7	Hind limb tremors when suspended by the tail
6	Weakness of one hindlimb extension when suspended by the tail
5	Weakness of two hindlimbs extension when suspended by the tail
4	One proximal hindlimb paralysed and walking on tiptoes
3	Two proximal hindlimbs paralysed and walking on tiptoes
2	Dragging one rigid hind limb
1	Dragging two rigid hind limbs
0	Unable to right itself within 30 s

B

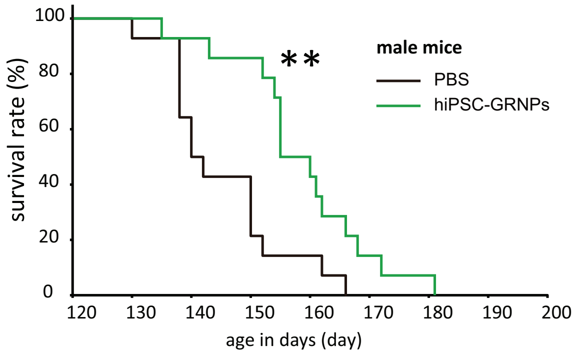

C

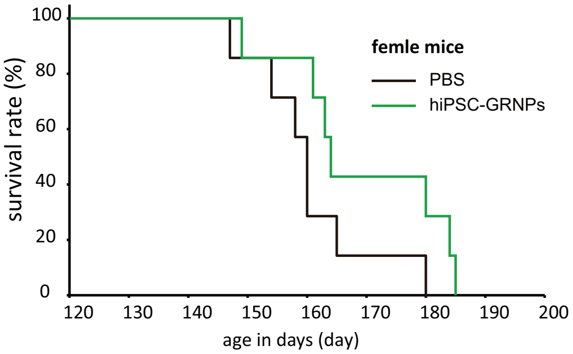

Figure S1, related to Figure 2. Separate evaluations of survival of male and female mice

(A) Motor function scoring system. (B) Kaplan-Meier plot of male ALS mice shows a significant difference between the PBS injection group survival (black line) and the hiPSC-GRNPs transplantation group survival (green line) throughout the course of the study (n = 14 mice per group, P = 0.00479 stratified log-rank test), suggesting that the hiPSC-GRNPs transplantation group had better survival (**p < 0.01). (C) Kaplan-Meier plot of female ALS mice do not show any significant difference between the PBS injection group survival (black line) and the hiPSC-GRNPs transplantation group survival (green line) throughout the course of the study (n = 7 mice per group, P = 0.11363 stratified log-rank test).

Figure S2

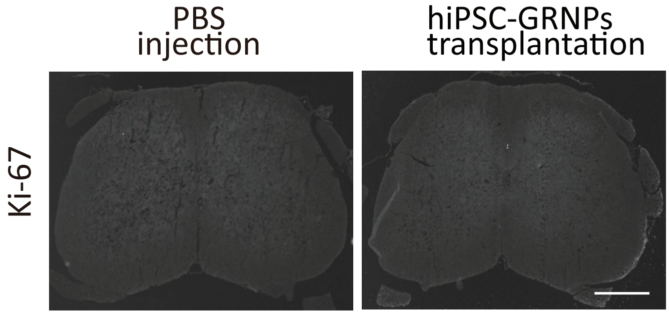

Figure S2, related to Figure 3. Histological evaluation for tumorigenicity

Tumorigenicity after transplantation was evaluated by immunostaining for Ki-67, a marker of proliferating cells. Tumor-like graft mass was not observed. No Ki67-positive grafts were found. Scale bar = 500 μm.

Figure S3

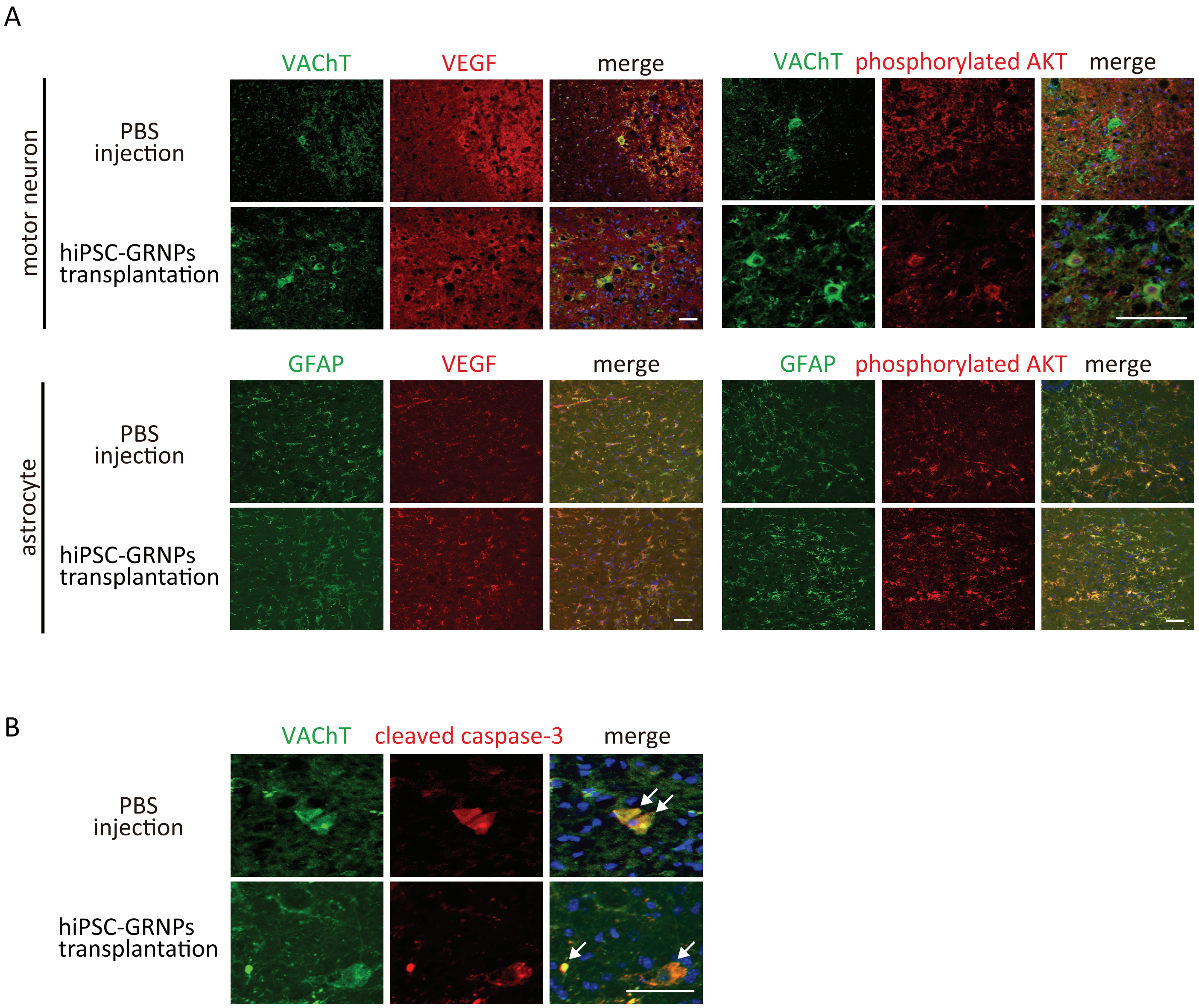
A
VAChT
VEGF
merge
VAChT
phosphorylated AKT
merge
motor neuron
PBS injection
hiPSC-GRNPs transplantation
GFAP
VEGF
merge
GFAP
phosphorylated AKT
merge
astrocyte
PBS injection
hiPSC-GRNPs transplantation
B
VAChT
cleaved caspase-3
merge
PBS injection
hiPSC-GRNPs transplantation

Figure S3, related to Figure 4. Histological evaluation of the effect of transplantation

(A) VEGF and phosphorylated AKT, and (B) cleaved caspase-3 were immunostained. VAChT and GFAP are motor neuron and astrocyte markers, respectively. Scale bars = 50 μm.

Table S1, related to Figure 2. Comparison with previous transplantation research with ALS model animals

study no.	graft type	host	transplantation site and condition	TP day	control	immunosupression	results and mechanistic insights	sex comparison	reference
1	human, NPC (22-week fetus) secreting human GDNF	rat	lumbar 12 x10^4 cells in 2 µl by glass capillary	90-100	vehicle solution	cyclosporine A	no benefit on survival increased ChAT+ soma size	not done	Klein SM et al. Hum Gene Ther. 2005 16(4):509-21.
2	human, NSC (8-week fetus)	rat	lumbar 5 x10^4 cells in 1 µl by glass capillary	62	dead cells	FK-506	improved survival upregulation of GDNF/BDNF	not done	Xu L et al. Transplantation. 2006 15;82(7):865-75.
3	mouse, NSC (6~8-week fetus)	mouse	lumbar 1 x10^4 cells in 1 µl by glass capillary	70	vehicle solution	not used	improved survival upregulation of VEGF/IGF1	not done	Corti S et al. Brain. 2007 130(5):1289-305.
4	mouse, olfactory bulb NSC	mouse	lumbar 500 neurospheres in 2 µl by steel needle	70	vehicle solution	not used	improved survival graft axons in ventral root	not done	Martin LJ et al. Neuropathol Exp Neurol. 2007 66(11):1002-18.
5	human, NPC (10~15-week fetus) secreting human GDNF	rat	lumbar 18 x10^4 cells in 2 µl by glass capillary	70	vehicle solution	cyclosporine A	prevented motor neuron death	female only	Suzuki M et al. PLoS One. 2007 1;2(8):e689.
6	rat, GRPs (2-week fetus)	rat	cervical 15 ×10^4 cells in 2 µl by 30G needle	90	vehicle solution or dead cells	cyclosporine A	improved survival upregulation of GLT1 decreased microgliosis	not done	Lepore AC et al. Nat Neurosci. 2008 ;11(11):1294-301.
7	human, GRPs (17~24-week fetus)	mouse	cervical 5 x10^4 cells, 2 µl by 30G needle	50-60	fibroblast	cyclosporine A	no benefit	not done	Lepore AC et al. PLoS One. 2011;6(10):e25968.
8	human, GRPs (17~24-week fetus)	mouse	cervical 5 x10^4 cells, 2 µl by 30G needle	50-60	fibroblast	FK-506 rapamycin	no benefit	not done	Lepore AC et al. PLoS One. 2011;6(10):e25968.
9	human, GRPs (17~24-week fetus)	mouse	cervical 15 x10^4 cells, 2 µl by 30G needle	50-60	fibroblast	FK-506 rapamycin	no benefit	not done	Lepore AC et al. PLoS One. 2011;6(10):e25968.
10	human, NSC (8-week fetus)	rat	cervical and lumbar 2 x10^4 cells in 1 µl by 33G needle	62	dead cells	FK-506	improved survival	not done	Xu L et al. Neurosci Lett. 2011 2;494(3):222-6.
11	human, NSC (fetus)	rat	lumbar 1 x10^4 cells in 0.5 µl by glass capillary	60-65	vehicle solution	FK-506 mycophenolate mofetil	transiently improved hind limb motor score no benefit on survival	no significance in analyzing males and females separately	Hefferan MP et al. PLoS One. 2012;7(8):e42614.
12	human, iPSC derived NSC	mouse	into tail vein 100 x10^4 cells in 100 µl	90	vehicle solution	not used	improved survival decreased microgliosis/astrogliosis	not done	Nizzardo M et al. Hum Mol Genet. 2014 15;23(2):342-54.
13	human, iPSC derived NSC	mouse	intrathecal 100 x10^4 cells in 5 µl	90	vehicle solution	not used	improved survival decreased microgliosis/astrogliosis	not done	Nizzardo M et al. Hum Mol Genet. 2014 15;23(2):342-54.
14	human, iPSC derived GRNPs	mouse	lumbar 4 x10^4 cells in 0.5 µl by 35G needle	90	vehicle solution	FK-506	improved survival upregulation of VEGF/phosphorylated AKT	significant only in male	this study

Vehicle solution includes PBS, saline and culture medium.
Abbreviation: NPC, neural progenitor cells; NSC, neural stem cells; GRP, glial-restricted progenitors; GDNF, glial cell line derived neurotrophic factors; TP, transplantation

Table S2, related to Figure 4. Primer names and sequences used in species-specific qPCR

Primer name	Forward	Reverse	Target Species	Amplicon Position
**rt_VEGF_Hu022**	cattggagccttgccttg	atgattctgccctcctcctt	Human	Exon1/2
**rt_VEGF_Ms022**	actggaccctggctttactg	tctgctctccttctgtcgtg	Mouse	Exon1/2
**rt_NGF_Hu032**	tccggacccaataacagttt	catggacattacgctatgcac	Human	Exon2/3
**rt_NGF_Ms096**	tatactggccgcagtgaggt	ggacattgctatctgtgtacgg	Mouse	Exon2/3
**rt_NTF3_Hu018**	gcgacaacagagacgctaca	cacgtaatcctccatgagatacaa	Human	Exon3
**rt_NTF3_Ms091**	ggaggaaacgctatgcagaa	gtcacccacaggctctcact	Mouse	Exon3
**rt BDNF_Hu054**	cggaaggacctatgtttgct	tatttcagaacgcgcaactg	Human	Exon2
**rt_BDNF_Ms031**	gtggtgtaagccgcaaaga	aaccatagtaaggaaaaggatggtc	Mouse	Exon2
**rt_HGF_Hu056**	gaaggatcagatctggttttaatga	tgcatccataattaggtaaatcaatc	Human	Exon15/16
**rt_HGF_Ms033**	gcgcaagcagatcttaaaca	aagttatccaggattgcaggtc	Mouse	Exon15/16
**rt_GDNF_Hu006**	agctgccaacccagagaat	aaatgtattgcagttaagacacaacc	Human	Exon3
**rt_GDNF_Ms026**	cctcgaagagagaggaatcg	cgacctttccctctggaat	Mouse	Exon3

Supplemental Experimental Procedures

GFP-labeled iPSC generation

Human iPSC 201B7 was cultivated on an SNL feeder cell layer in primate embryonic stem cell medium (ReproCELL Inc., Japan) supplemented with 4 ng/ml basic FGF (Wako Pure Chemicals Industries, Ltd, Japan). The medium was changed every day. The *piggyBac* transposon vector (PB-EF1alpha-EiP) was constructed to express EGFP and puromycin resistance gene under control of the human EF1α promoter. We transfected 201B7 human iPS cells with PB-EF1alpha -EiP and *piggyBac* transposase expressing plasmids by using FuGENE HD (Promega) reagent on Matrigel (Becton Dickinson)-coated plates. Puromycin selection (1 μg/ml) was applied from 3 days after transduction, and was maintained for several passages to ensure stable and long-term expression of EGFP. After GFP-labeling and puromycin selection, 46XX karyotype was confirmed by Mitsubishi Chemical Medience Corporation.

Preparation of hiPSC-GRNPs for transplantation

hiPSCs were dissociated to single cells and quickly reaggregated in U-bottom 96-well plates (Greiner bio-one, Germany), pre-coated with 2% Pluronic F-127 (Sigma-Aldrich) in 100% ethanol, for suspension culture. Aggregations, EBs, were cultured in ‘DFK5% medium’ (DFK5%; DMEM/Ham’s

F12 (Gibco) supplemented with 5% KSR (Gibco), NEAA (Invitrogen, Japan), L-glutamine (Sigma-Aldrich), 0.1 μM 2-mercaptoethanol (Invitrogen)) with 2 μM dorsomorphin (Sigma-Aldrich) and 10 μM SB431542 (Cayman Chemical, U.S.A.) during the neural inductive stage (day 0 to 8). After the neural-induction stage, EBs were transferred onto Matrigel (Becton Dickinson)-coated 6-well culture plates and cultured in DFK5% supplemented with 1x N2 supplement (Invitrogen), 10 ng/ml human BMP4 (R&D, USA), and 10 ng/ml human LIF (R&D) during the patterning stage (day 8 to 28). Large numbers of neural precursor cells (NESTIN-positive) were observed to migrate from the edge of EBs. At day 28 *in vitro*, migrated neural precursor cells were harvested from the plates using Accutase (Innovative Cell Technologies, Inc.), centrifuged (200 g, 5 min), and resuspended in sterile PBS, without the addition of any neurotrophic factors at a concentration of 80,000 cells/μl. After completion of the transplantation, we evaluated the viability of hiPSC-GRNPs by trypan-blue exclusion assay. Viability was always more than 90%.

Transplantation

The mice were anesthetized with pentobarbital sodium (20 mg/kg; in room air, Kyoritsuseiyaku Corporation) and placed into a spinal clamp apparatus (Narishige, Japan). Muscle and connective tissue were dissected, through the lamina of the Th12/L1 vertebrae, which corresponded to the L3-L4 segment

of the spinal cord. Each mouse received two grafts (bilaterally at L3-L4) of 4 x 10^4 cells (in 0.5 μl PBS) into the ventral horn. Cells were delivered by 10-μl Gastight syringe (Hamilton, Sigma, Japan) with a 35-gauge/25-degree beveled needle (React Systems, Osaka, Japan). The needle tip was positioned at 150 μm laterally from the mid-line, lowered to a depth of 1,000 μm below the surface of the spinal cord and was held in place for 2 min both before and after cell injection. Cells were delivered under the control of a microsyringe pump controller, Nanojet (CHEMIX, Stafford, USA) at a rate of 0.1 μl/min. Control animals were injected with 0.5 μl of PBS solution per horn in a similar fashion as the cell-transplanted animals. Tg-G93A SOD1 mice were divided into hiPSC-GRNPs transplantation (n = 24 total: n = 17 males, n = 7 females) and PBS injection group (n = 24 total: n = 17 males, n = 7 females). The study was designed such that littermates were distributed equally among the transplanted and non-transplanted groups. Immunosupressant (FK506 3 mg/kg/day; Astellas, Tokyo, Japan) was administered orally to both PBS injection and hiPSC-GRNPs transplantation groups, from one week before the operation for the duration of the study. For axon count, two groups of animals (each consisting of n = 3 males) were sacrificed at 120 days of age and excluded from survival assessment.

Animal care

All animals were cared for and procedures performed in accordance with the Kyoto University

Animal Institutional Guidelines and were maintained in a specific pathogen-free environment. All animal experiments were approved by the CiRA Animal Experiment Committee (No. 24 and No. 27). The ALS model mouse line (B6.Cg-Tg(SOD1*G93A)1Gur/J (Tg-G93A SOD1 mouse)) was obtained from Jackson Laboratories and Oriental BioService. Animals were housed under a light:dark (12:12h) cycle and provided with food and water *ad libitum*.

Assessment of neurological function and disease progression

Motor function was evaluated by clinical grading system (Figure S1A) (Zhou et al., 2007) and scored by two experimenters blinded to the treatment groups. To determine disease end-stage in a reliable and ethical fashion, end-stage was defined by the inability of mice to right themselves within 30 seconds when placed on their sides.

Immunohistochemistry

Mice were anesthetized with pentobarbital and perfused transcardially with ice-cold phosphate buffer saline (PBS, pH 7.4) and subsequently with 4% paraformaldehyde in PBS. All mice were perfused under identical conditions by the same individual. Tissues were then cryoprotected in a 20% sucrose solution in PBS at 4°C until they sank. They were frozen in Tissue-Tek O.C.T. Compound (Sakura

Finetek, Japan). We confirmed the injection sites by stereo microscope (Leica, Japan) and cut the lumbar spinal cord at injection sites to freeze samples in Tissue-Tek O.C.T. Compound. The spinal cords were sliced into 10-μm transverse sections on a cryostat at −20°C, and every tenth section from the injection sites was mounted on slide glass to be stored at −80°C until use. Tissue sections were permeabilized in PBS containing 0.1% Triton X-100 for 15 min at room temperature, followed by rinsing with PBS. Nonspecific binding was blocked with PBS containing 10% donkey serum for 60 min at room temperature. Slices were incubated with primary antibodies overnight at 4°C, and then labeled with appropriate fluorescent-tagged secondary antibodies. DAPI (Life Technologies) was used to label nuclei. Fluorescence images were acquired on a BioRevo fluorescent microscope (Keyence, Osaka, Japan), LSM710 microscope (Carl Zeiss, Göttingen, Germany), or In Cell Analyzer 6000 (GE Healthcare, Japan). The following primary antibodies were used in immunocytochemistry or immunohistochemistry: MAP2, microtubule-associated protein 2 (1:2,000; Millipore, Japan), GFAP, glial fibrillary acidic protein (1:2,000; DAKO, Japan), GLT-1, glutamate transporter 1 (1:200; Abnova, USA), ALDH1L1, aldehyde dehydrogenase 1 (1:400; Abnova), NESTIN (1:2,000; Millipore), TUJ1 (1:4,000; Millipore), A2B5 (1:400; Millipore), CNPase (1:400; Cell Signaling Technology, Japan), VAChT (1:400; Chemicon, Japan), cleaved caspase-3 (1:400; Cell Signaling Technology), phosphorylated AKT (1:400; Cell Signaling Technology), GFP, green fluorescence protein (1:3,000; Invitrogen) and Iba1 (1:400; Wako

Pure Chemicals Industries, Ltd., Japan). For evaluating the positive count ratio of immunocytochemistry, we imaged the cells in the ventral horn parenchyma using automated microscopy, IN Cell Analyzer 2000 or 6000 (GE Healthcare, Japan), and counted the immunostained structural components by using IN cell developer toolbox software (GE Healthcare).

Morphological analysis of axons

Lumbar roots, postfixed in 4% paraformaldehyde/PBS, were frozen in Tissue-Tek O.C.T. Compound (Sakura Finetek) and transversely sectioned into 5-μm slices at −20°C and stored at −80°C until use. Sliced lumbar roots were stained with neurofilament-H antibody (1:2,000; Millipore). Entire roots were imaged with a BioREVO fluorescent microscope. Axonal diameters of L4 roots were measured by ImageJ software and grouped into 1-μm bins.

Human- or mouse-specific quantitative RT-PCR for spinal cord

Total RNA from the injection sites of mouse lumbar spinal cords was extracted by RNeasy plus kit (QIAGEN). Five-hundred nanograms of total RNA was reverse-transcribed into cDNA using RevaTra Ace with random primers (Toyobo, Osaka, Japan). To analyze separate gene expressions from graft or host origin, we designed human- or mouse-specific PCR primers specific for VEGF, vascular endothelial

growth factor; NGF, nerve growth factor; NT3, neurotrophin-3; BDNF, brain-derived neurotrophic factor; GDNF, glial cell-line derived neurotrophic factor; and HGF, hepatocyte growth factor (Table S2). One μL of the generated cDNA was used as template for each reaction in real-time quantitative PCR analysis, using SYBR Green II (TAKARA, Shiga, Japan) and StepONE plus (Life Science Technology). GAPDH was used as endogenous control gene to normalize target genes. To perform relative quantification, the comparative threshold (Ct) cycle method was used. The fold change in gene expression profile was referred to a PBS-injection group.

Western blot analysis

Tissues, removed from mice, were lysed in RIPA buffer (50 mM Tris-HCl buffer, pH 8.0, 150 mM NaCl, 1% NP-40, 0.5% deoxycholate, 0.1% SDS, and protease inhibitor cocktail (Roche Diagnostics)). The lysates (20 μg/lane) were subjected to SDS-PAGE and then electrically blotted to a polyvinylidene difluoride sheet. The sheet was soaked with an appropriate first antibody and subsequently with HRP-labeled anti-mouse IgG or anti-rabbit IgG (Bio-Rad). The following primary antibodies were used in western blot analysis: total AKT (1:3,000; Cell Signaling Technology), phosphorylated AKT (1:3,000; Cell Signaling Technology), VEGF (1:3,000; Abcam) and β-Actin (1:20,000; Sigma). The antigenic bands were visualized with ECL prime (GE Healthcare). The images were acquired on LAS 4000 (GE

Healthcare). The intensity of the protein band was analyzed using ImageQuant TL software (GE Healthcare).

Statistical analysis

We used JMP® 9 software for statistical calculation and drawing survival time graphs (SAS Institute Inc., Cary, NC, USA).

Supplemental reference

Zhou, C., Zhao, C.P., Zhang, C., Wu, G.Y., and Xiong, F. (2007). A method comparison in monitoring disease progression of G93A mouse model of ALS. Amyotroph Lateral Scler *8*, 366-372.
